# A Review on the Rheological Properties of Single Amino Acids and Short Dipeptide Gels

**DOI:** 10.3390/gels10080507

**Published:** 2024-08-01

**Authors:** Sérgio R. S. Veloso, Mariangela Rosa, Carlo Diaferia, Célio Fernandes

**Affiliations:** 1 Physics Centre of Minho and Porto Universities (CF-UM-UP), Laboratory of Physics for Materials and Emergent Technologies (LaPMET), University of Minho, Campus of Gualtar, 4710-057 Braga, Portugal; sergioveloso96@gmail.com; 2 Department of Pharmacy, Centro Interuniversitario di Ricerca sui Peptidi Bioattivi “Carlo Pedone” (CIRPeB), University of Naples “Federico II”, Via Tommaso de Amicis 95, 80131 Naples, Italy; mariangela.rosa@unina.it (M.R.); carlo.diaferia@unina.it (C.D.); 3 Transport Phenomena Research Centre (CEFT), Department of Mechanical Engineering, Faculty of Engineering, University of Porto (FEUP), Rua Dr. Roberto Frias s/n, 4200-465 Porto, Portugal; 4 Centre of Mathematics (CMAT), School of Sciences, University of Minho, Campus of Gualtar, 4710-057 Braga, Portugal

**Keywords:** peptide hydrogels, peptide materials, amino acid hydrogels, mechanical properties, self-assembly

## Abstract

Self-assembled peptide-based hydrogels have attracted considerable interest from the research community. Particularly, low molecular weight gelators (LMWGs) consisting of amino acids and short peptides are highly suitable for biological applications owing to their facile synthesis and scalability, as well as their biocompatibility, biodegradability, and stability in physiological conditions. However, challenges in understanding the structure–property relationship and lack of design rules hinder the development of new gelators with the required properties for several applications. Hereby, in the plethora of peptide-based gelators, this review discusses the mechanical properties of single amino acid and dipeptide-based hydrogels. A mutual analysis of these systems allows us to highlight the relationship between the gel mechanical properties and amino acid sequence, preparation methods, or N capping groups. Additionally, recent advancements in the tuning of the gels’ rheological properties are reviewed. In this way, the present review aims to help bridge the knowledge gap between structure and mechanical properties, easing the selection or design of peptides with the required properties for biological applications.

## 1. Introduction

**Figure 1:**
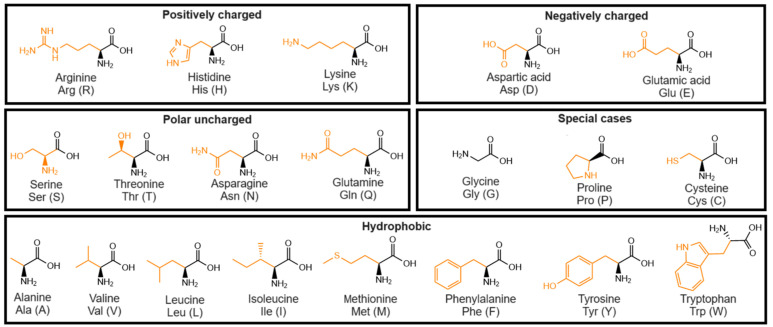
Structure of the amino acids that constitute the building blocks of peptides.

Hydrogels consist of three-dimensional networks of cross-linked chains in which a substantial amount of water is entrapped. Among the several materials, self-assembled peptide-based hydrogels have gained significant attention in recent years for biomedical applications, including drug delivery [1], wound healing [2], bioimaging [3], biosensors [4], and tissue engineering [5]. This can be attributed to the suitable properties of peptide-based gels, such as low immunogenicity, biocompatibility, biodegradability, low toxicity, high water content, and stability in physiological conditions [6,7,8]. In particular, low molecular weight gelators (LMWGs) have attracted attention due to their straightforward synthesis, high scalability, cost-effective production, spontaneous self-assembly, responsiveness to stimuli, and ease of injectability [9]. These compounds, consisting of amino acids (Figure 1) and small peptides, constitute the building blocks that self-assemble into anisotropic structures, which, under appropriate conditions, interact (through entanglement or cross-linking) to form a gel matrix [10,11]. 

**Figure 2:**
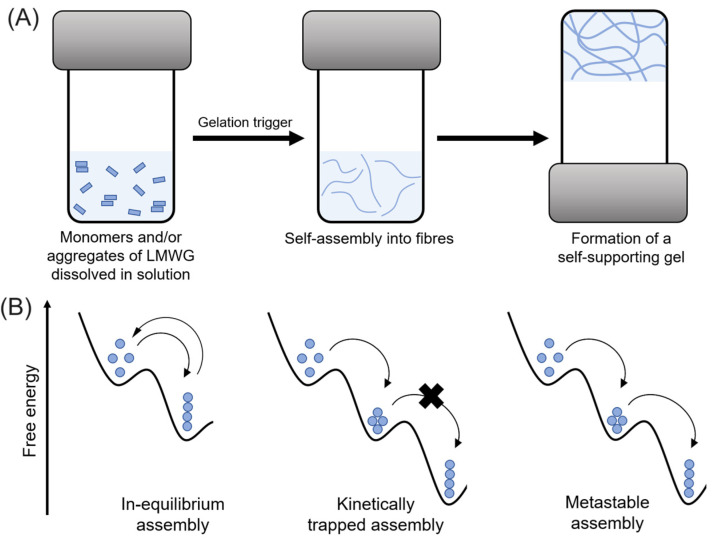
(**A**) Schematic representation of LMWG self-assembly into a hydrogel. The LMWGs may initially exist as monomers and/or aggregates. Upon triggering, the LMWGs self-assembles into hierarchical anisotropic structures, such as fibrils. These structures further develop into entangled/crosslinked mature fibers, entrapping the solvent and forming a gel. (**B**) Schematic representation of the possible self-assembly energy landscapes. In “in-equilibrium assembly”, the assembled peptides can easily reach a low-energy state due to the fast exchange of building blocks, enabling rearrangement into a more favorable state. However, since self-assembly is usually a high-rate process, kinetically trapped structures are often formed rather than the most stable state. Additionally, the negligible exchange of molecules trapped in a local energy minimum prevents rearrangement into a more favorable state. Thus, kinetically trapped structures may reside in sufficiently deep energy wells, resulting in stable gel states. However, if there is some exchange with the environment, a slow rearrangement into a thermodynamically favored state can occur, which is referred to as a metastable assembly.

The assembly process relies on the synergistic effect of non-covalent interactions, including hydrogen bonding, van der Waals forces, electrostatic interactions, and hydrophobic and/or aromatic π-π interactions [12,13]. Consequently, the amino acid sequence can strongly influence the self-assembly properties, making it a challenging task to predict gel formation for a given peptide structure [14,15,16,17,18,19,20,21,22]. Generally, gel formation is straightforward and can be induced by different stimuli such as adjusting the pH, heating/cooling, exposure to light, solvent switch, sonication, enzymatic reactions, and the addition of (metal) salts to a solution or dispersion of gelator molecules (Figure 2A) [23,24]. Additionally, because the self-assembly process is too rapid for the structures to achieve their targeted or global thermodynamic minimum, the self-assembled structures commonly consist of kinetically-trapped or metastable states (Figure 2B) [13,25]. 

Hereby, the gel properties are significantly affected by the self-assembly pathway [13,23,26,27], which encompasses various factors such as gelling temperature, cooling rate during thermal annealing, type and concentration of salt, final pH, rate of pH change, and the solvent used [23,28,29,30,31,32]. Thus, the self-assembly conditions can lead to different shapes, including micelles, vesicles, and nanotubes, in addition to fibers [33,34,35,36]. The properties can be further controlled by combining with other peptide (non-)gelators, known as multicomponent gels [29,37,38,39,40], or by including nanomaterials to obtain composite gels [41,42,43]. Additionally, depending on the peptide structure, the resulting hydrogels can exhibit stimuli-responsiveness (pH, temperature, light, ionic strength, mechanical), sol–gel transition, thixotropy, and the capability to entrap drugs through physical or chemical linkage [28,44,45]. Among these, thixotropic gels are particularly important in various biomedical fields. Thixotropy involves fast shear thinning and recovery, associated with the reversible breaking and recovery of the network after mechanical disruption owing to the non-covalent interactions between LMWGs [44]. However, only a few peptide gelators display this property, and its association with the structure is still not understood. Comprehensive reviews were carried out by Zanna et al. [44] and Pramanik [9], in which the thixotropic peptide gels are noted to commonly display a storage moduli of 10^2^–10^3^ Pa, which is a suitable range for application in biological soft tissues [46,47].

Therefore, understanding the relation between peptide structure and gelation capability, along with the thixotropic behavior, remains a challenging task. Moreover, comprehending the structure–mechanical properties relationship is crucial for designing peptides with the desired mechanical behavior for various biological applications.

In this way, this review focuses on the mechanical properties commonly exhibited by different peptide structures, emphasizing the studies on self-assembly and mechanical properties. The rheological properties of gels derived from single amino acids and dipeptides are discussed. Specific and characteristic gel features (including preparation approach, pH, applications, or supramolecular elements) are summarized in Tables. Furthermore, recent advancements in tuning the rheological properties for different biological applications are reported, and current challenges are discussed. This review aims to help bridge the knowledge gap concerning the structure–property relationship, thereby contributing to the design of new peptides for biological applications.

## 2. Rheological Characterization Techniques

Rheological characterization is widely used to study the mechanical properties of bulk hydrogels, such as changes in the mechanical behavior induced by stimuli, the effect of combining different peptides or incorporating composites, and the gel’s ability to recover after experiencing shear flow or a large strain. Common rheometric experiments include monitoring the storage (G′) and loss (G″) moduli as a function of time (oscillatory time sweep), applied angular frequency (frequency sweep), and applied oscillatory strain (strain sweep measurements), which are commonly measured by bench-top rheometers. As a rule of thumb, a gel is generally characterized by a lack of frequency dependence in a frequency sweep and a G′ value nearly an order of magnitude greater than G″ (G′ >> G″), mainly at low frequencies [48,49,50]. For instance, a material may exhibit solid-like behavior (G′ >> G″) at high frequencies (short time scales) but behave more like a liquid (G″ > G′) at low frequencies (long time scales). Other techniques include micro-rheology [51,52], cavitational rheology [53,54], and large amplitude oscillatory dilation (LAOD) to study interfaces [55]. 

Strain sweep measurements are employed to determine the linear viscoelastic regime (LVR) for a given material, which, along with frequency sweep, should be among the first steps in the rheological characterization of a hydrogel [49]. Posteriorly, G′ and G″ values can be measured during an oscillatory time sweep at constant frequency and strains within the LVR, providing information about the mechanism and kinetics of network formation (e.g., during the initial gelation or recovery after a large strain). Additionally, nonlinear rheological measurements, such as large-amplitude oscillatory strain (LAOS) tests, are commonly performed to gain further insight into the hydrogel network. These studies are crucial for developing tissue engineering scaffolds and cell culturing substrates since scaffolds’ mechanical properties should closely match the healthy tissue or extracellular matrix to ensure cellular viability, differentiation, proliferation, and migration, among other cellular processes [56,57,58].

As mentioned, rheology has been commonly employed to study the gelation of peptide-based gels [59,60]. Chen et al. [61] demonstrated that various functionalized dipeptides (including naphthalene rings and alkyl chains) could form gels at pH 11.7 through the addition of Ca^2+^, as well as trivalent and polyvalent salts. The viscosity was found to correlate with the peptide’s hydrophobicity and to be aging dependent, with *N*-capped diphenylalanine forming gels with G′ values near 10^4^–15^5^ Pa. The gels could recover after disruption but were not thermoreversible. Interestingly, the structures that afforded viscous solutions in the range of 40–60 mPa s^−1^ could form the strongest gels. 

In short peptides, the magnitude of the stimulus usually influences the self-assembly and sometimes the resulting hydrogel mechanical properties [26,60,62,63]. Gels prepared with glucono-δ-lactone (GdL), which provides a slower pH decrease than HCl, demonstrate improved homogeneity and mechanical properties (in which G′ values can increase more than an order of magnitude) [64,65,66,67,68]. Temperature enhances the hydrolysis rate of GdL to gluconic acid, affecting the pH decreases and kinetics of self-assembly. Cardoso et al. [63] reported similar mechanical properties regardless of the gelation temperature, though other works reported significant changes in the gelation kinetics [26,69]. Higher enzyme content in enzyme-triggered gels has commonly resulted in faster gelation and larger G′ values [70,71,72,73], which can also be achieved with higher ionic force [60,62,74,75,76]. For example, Stendahl et al. [62] reported a storage modulus increase of over three orders of magnitude with increasing Ca^2+^ content and cation valence as follows: M^+^ < M^2+^ (*s* block) < M^3+^ (*d*, *p*, *f* block) < M^2+^ (*d* block), forming gels with G′ values up to ~10^4^ Pa. The authors analyzed this dependence based on the Irving–Williams series for ionic binding affinity, with d-block ions displaying greater potential for coordination bonding and forming stronger gels. Veloso et al. [69] observed faster gelation kinetics and lower storage modulus for dehydropeptide-based gel prepared at higher temperatures. Other parameters, such as concentration and pH, can also strongly influence the mechanical properties [77].

Despite similar G′ and G″ values and large frequency independence, gels produced by different methods may exhibit differences in the strain sweeps. Therefore, it is important to conduct a comprehensive rheological characterization of the gels. For example, Colquhoun et al. [78] reported that gels prepared through pH- and solvent-switch showed similar frequency sweep profiles, but the pH-induced gels broke sharply at low strain, while the solvent-switch gels exhibited a gradual decrease at larger strains without moduli crossover. In addition, evaluating the gel’s heterogeneity is important to ensure the reproducibility of the gel preparation method, which cannot be determined from bulk rheological measurements. Alternatively, this can be probed with cavitation rheology, which involves monitoring the pressure dynamics of a bubble (or cavity) pumped into a gel [49,54,79]. Measuring the maximum pressure sustained by the internal void allows for comparing different gels and probing local points to assess the mechanical homogeneity and/or gradients, enabling the study of layer-by-layer structures.

The exponent x of the G′ dependence on concentration c, G′ ≈ c^x^, has been associated with a specific network type in various studies [80,81,82,83,84,85,86,87,88]. However, different networks have shown similar values of x [88,89], making it unclear how x relates with the network type. Multicomponent gels further complicate rheological characterization, as gelators can co-assemble or form self-sorted structures that interact with each other [50,53]. These systems can display increased [90,91,92], similar, or weaker stiffness [93,94] compared to single components. A stronger multicomponent gel may result from more gelators in the system or new fiber formation that increases stiffness. A stiffer gel can be achieved through more cross-links [91] or favorable interactions between fibers in a self-sorted system [92]. Conversely, a similar or weaker stiffness than single-component gels can result from a weaker second network, weaker or less entangled co-assembled fibers, or unfavorable interactions in self-sorted fibers, which can include steric crowding. Thus, the G′ and G″ values are insufficient to determine the assembly type, such as whether it is a self-sorted or co-assembled system. Lyanage et al. [95] reported this lack of correlation between the assembly type and mechanical properties for several self-assembled/coassembled Fmoc-protected phenylalanine derivatives. Additionally, the causes of the changes in mechanical properties are difficult to deconvolute, such as in the case that the multicomponent gel displays both a larger storage modulus and faster kinetics [96]. Generally, more homogenous gels have better mechanical properties, but LMWGs can also gel quickly due to higher cross-linking numbers, leading to more inhomogeneities. Nonetheless, network changes can be identified through differences in rheological properties, such as in the strain sweeps [97,98] or time sweeps [99,100]. These changes may include a multi-step breakdown behavior, sharp break or creaming behavior, a large strain to break the gel network, or the gelation exhibiting a multi-step increase in G′ and G″ [53]. However, determining the network type remains a challenging task. For example, Cornwell et al. [101] showed that one gelator could assemble by lowering the pH, and a second component could assemble under UV irradiation, resulting in a self-sorted system. However, it remains unclear whether this consists of an interpenetrating network or if the second network grows on the first. Therefore, rheology can provide insight into the dynamics and changes of gel systems, but offers limited information on the network morphology and structure, particularly in multicomponent gels.

## 3. Overview of the Mechanical Properties of Single Amino Acids and Dipeptide-Based Gels

### 3.1. Single Amino Acids

#### 3.1.1. Uncapped Amino Acids

**Figure 3:**
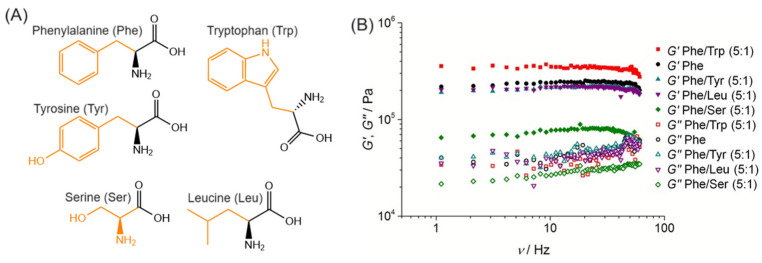
(**A**) Structure of the amino acids used for the formation of co-assembled gels, and the respective (**B**) storage (G′) and loss (G″) moduli at increasing frequency sweeps. The figure is adapted from Ref. [104]. Copyright with permission from John Wiley and Sons.

Shorter peptides lower the production cost and facilitate the scalability of production. Among the amino acid-based gels, phenylalanine stands out in the development of new hydrogels. Both the L- and D- uncapped phenylalanine are reported to form gels upon a heating/cooling cycle in a wide pH range [102]. These gels consist of a polymorphic system comprising both crystals and gel phases, in which the properties can be tailored by co-assembly with non-gelling additives [103,104]. However, co-assembling both enantiomers is described as not yielding gels, forming flake-like structures that phase out from solution [102]. The uniqueness of phenylalanine is further highlighted by the inability of alpha-phenylglycine to form fibrous gels [102], making phenylalanine the smallest known amino acid capable of forming gels. These gels are thermoreversible, and their rheological properties can be tuned by co-assembling with other amino acids such as leucine, serine, tryptophan, and tyrosine [104]. Importantly, phenylalanine gels display G′ values in the order of 10^5^ Pa (~2.0 × 10^5^ Pa), nearly twice larger than G″ values (~4.5 × 10^4^ Pa), in which significant changes could be obtained by mixing with tryptophan (increasing G′ to ~3.5 × 10^5^ Pa) or serine (decreasing G′ to ~7.5 × 10^4^ Pa) (see Figure 3). The self-assembly of other (non-)aromatic amino acids has also been explored, resulting in different nanostructures, including nanotubes, nanoribbons, nanosheets, etc., but these structures do not form gels [105,106,107].

#### 3.1.2. Fluorenylmethyloxycarbonyl N-Capped Phenylalanine (Fmoc-Phe) and Derivatives

Fmoc-AAs are among the most widely studied low-molecular-weight gelators, benefitting from their wide commercial availability. Gels with either a single or a mixture of Fmoc-AAs have been reported [108,109,110,111]. These LMWGs have also been combined with composites such as graphene oxide, carbon nanotubes, and silver nanoparticles to improve the tunability of the gel properties and increase the storage modulus of the native hydrogel [112,113,114]. However, as in other supramolecular gels, the self-assembly pathway can strongly affect the mechanical properties. For example, Fmoc-Phe can form gels through a heating/cooling cycle [113,114], slow pH decrease by adding glucono-δ-lactone [115,116], and solvent exchange [95,117]. As summarized in Table 1, varying the preparation method can affect the gel morphology, gel–sol temperature, and the storage modulus by up to three orders of magnitude. Furthermore, even a small change in the final pH after adding GdL can affect the critical strain. Hence, continuous efforts to improve the control of these properties have included modifications of the phenylalanine aromatic ring and the carboxylic acid. For instance, Nilsson et al. [108] reported that decreasing the electron density of the phenyl ring in the penta-fluorinated analog (Fmoc-F_5_-Phe) enabled the rapid formation of gels (~3–5 min) through solvent exchange, resulting in a high storage modulus (~3000 Pa), while Fmoc-Phe precipitated from solution. The same group also demonstrated that the penta-fluorinated and meta-fluorinated (Fmoc-3F-Phe) derivatives could form gels with thixotropic shear response for both slow pH decrease and solvent exchange [118]. Additonally, modifying the carboxylic acid with diaminopropane (DAP) led to fluorinated Fmoc-Phe derivatives that could assemble in physiologically relevant sodium chloride concentrations, assisted by a heating/cooling cycle, forming gels with larger storage modulus (~10^4^ Pa), shear thinning behavior, and enabling the sustained release of diclofenac [119]. 

**Figure 4:**
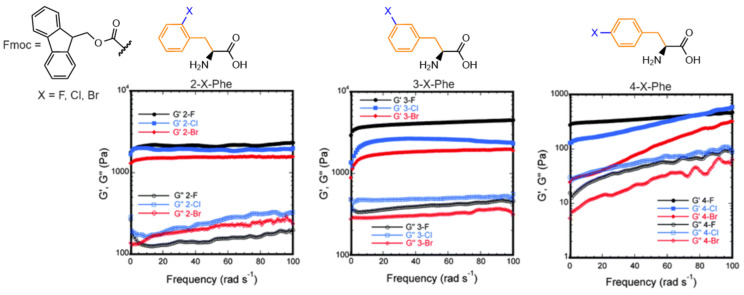
Oscillatory frequency sweep rheology of Fmoc-n-X-Phe hydrogels: Fmoc-2-X-Phe, Fmoc-3-X-Phe, and Fmoc-4-X-Phe. The figure is adapted from Ref. [133]. Copyright with permission from the Royal Society of Chemistry.

The steric effect and electronic character of halogen (F, Cl, Br) substitution at ortho, meta, and para positions were found to influence the resulting storage modulus in the order meta > ortho > para, while the halogen ion led to a decreasing rigidity in the order F > Cl > Br. Thus, a smaller and more electronegative halogen was associated with an increased storage modulus (Figure 4) [133]. The storage modulus could vary by one order of magnitude, from Fmoc-4-Br-Phe (~130 Pa) to Fmoc-3-F-Phe (~4200 Pa). However, the self-assembly rate did not correlate predictably with the halogen identity, following the order para > meta > ortho, with a faster gelation associated with a weaker gel, influenced by the interplay between electronic effects and steric perturbation.

Nilsson’s group also studied the influence of other substituents in the para position, including NO_2_, CN, F, NH_2_, OH, and CH_3_, in gels obtained through solvent exchange [95]. Concerning the kinetics of gelation, electron-withdrawing groups (such as NO_2_, CN, and F) facilitated a faster gelation process (ranging from 0.5 to 5 min) compared to Fmoc-Phe (25 min), and electron-rich derivatives (NH_2_, OH, CH_3_), which gelled within 10 min to 24 h. The observed trend in gelation rates followed the sequence NO_2_ > F > CN > CH_3_ > OH > NH_2_. This effect was attributed by the authors to a reduction in electrostatic repulsion between the aromatic π-systems of adjacent benzyl groups within the assembled fibrils, which aligns with the Hunter/Sanders electrostatic model. However, the electron-donating substituents generally displayed higher G′ values than the derivatives with electron-withdrawing substituents, with the order NH_2_ > OH > NO_2_ > CH_3_ > CN > F > H. Additionally, the gel rigidity decreased with both electron-donating (NH_2_ > OH > CH_3_) and electron-withdrawing (NO_2_ > CN > F) capabilities, indicating that both steric and electronic effects significantly influence the self-assembly process.

The C-terminus of fluorinated Fmoc-Phe gelators also plays a critical role in self-assembly and hydrogel formation, as demonstrated by Ryan et al. [134]. To evaluate the effect of the C-terminus, the carboxylic acid was converted to amide and methyl ester groups, modifying the hydrophobicity and hydrogen bond capability of the C-terminus. The C-terminal esters were observed to assemble into fibrils at a slow rate and were unable to form gels. In contrast, the amide derivatives assembled more rapidly than the parent carboxylic acids at both acidic and neutral pH. However, these amide-derived hydrogels were unstable under shear stress, a limitation attributed to the reduced water solubility of the amide group. Importantly, co-assembled gels of acid and amide functionalized monomers could form gels in phosphate-buffered saline (G′ ~100 Pa), while the parent acids could not, though more rigid co-assembled gels (G′ ~700–800 Pa) were obtained in un-buffered water, highlighting the complex role of the solvent interactions.

Fmoc-Tyr and its derivatives have also been explored as gelators, including the phosphorylated precursors to obtain gels upon enzymatic [70,121,135,136,137] or catalytic [138] dephosphorylation. Draper et al. [115] showed that the OH group in the para position induced different assemblies compared to Fmoc-Phe, with the latter forming metastable gels that easily crystallize with a similar phase, while Fmoc-Tyr displayed different fiber and crystal phases. The crystal phase was a result of the interactions between the planar Fmoc groups, while the hydrogen bonding drove the self-assembly into the fibrillar structures. Nevertheless, gels obtained through a heating/cooling cycle, slow pH decrease, and solvent switch have also been described, with the storage modulus varying nearly two orders of magnitude [95,115,120]. The substitution in the meta position with the electron-withdrawing NO_2_ led to thixotropic gels that could be prepared in a wide pH range (4.5–8.5) [122]. Similarly to Fmoc-Phe, co-assembled gels comprising Fmoc-Tyr were also developed to improve the mechanical properties and combined with composites, such as carbon nanotubes or graphene oxide, to enable photothermia upon near-infrared light irradiation [123]**.**

#### 3.1.3. Fluorenylmethyloxycarbonyl N-Capped Amino Acids (Fmoc-AAs)

Other Fmoc-AA hydrogels obtained through a heating/cooling cycle or slow pH decrease have also been reported in the literature, including Fmoc-Trp and non-aromatic amino acids, such as Fmoc-Met, Fmoc-Gly, and Fmoc-Ile, with storage moduli ranging from 10^2^ to 10^4^ Pa [115,120,139]. Interestingly, hydrogels based on Fmoc-Trp, Fmoc-Met, and Fmoc-Tyr were observed to selectively inhibit the growth of Gram-positive bacteria, following the order Fmoc-Trp > Fmoc-Met > Fmoc-Tyr [120]. Generally, non-aromatic amino acids are unable to form hydrogels, leading to other assemblies such as micelles and tubes [107,140]. However, attempts have been made to form hydrogels with these amino acids. For instance, Song et. [124] reported several Ag^+^-coordinated Fmoc-AAs hydrogels, including Fmoc-Pro, Fmoc-His, Fmoc-Leu, and Fmoc-Ala, with G′ values in the range ~10^2^–10^3^ Pa. These gelators could induce the detachment of the plasma membrane and consequent leakage of the cytoplasm upon interaction with the bacteria cell walls and membrane, leading to significant antibacterial effects in both Gram-negative (*Escherichia coli*) and Gram-positive (*Staphylococcus aureus*) bacteria. The co-assembly has also been proposed as a method to obtain gels with non-aromatic Fmoc-AAs. For example, Yang et al. [141] reported the formation of gels based on Fmoc-Leu and Fmoc-Lys through the addition of sodium carbonate, which could not form gels independently. Additionally, structural modification of the Fmoc-lysine side chain with biotin [125] and Fmoc group [126,142] was demonstrated to afford thixotropic hydrogels. Alternatively, non-proteinogenic amino acids, which are proteolytically unstable, were proposed by Arokianathan et al. [127]. These peptides included 2,3-diaminopropionic acid (Dap), a precursor in the synthesis of antibiotics such as viomycin and capreomycin, and were functionalized with Fmoc at both amino terminals. Hydrogels could be obtained across a wide pH range, with mechanical strength (in the range of 10^0^–10^2^ Pa) and thermal stability decreasing with increasing pH. Furthermore, the gels displayed thixotropy and could enhance cell proliferation at physiological pH (7.4).

#### 3.1.4. Other N-Capped Amino Acids

Despite advancements with Fmoc-AAs, concerns remain about the potential toxicity of polyaromatic cycles, requiring careful evaluation, as cell viability is negatively affected by Fmoc derivatives [143,144]. Consequently, other capping groups have been explored. Naphthalene is a particularly popular choice, with the fluorinated Phe derivates also displaying a larger storage modulus (G′ ~10^3^) than the parent compound (1-NapAc-Phe, G′ ~10^2^) [117]. Additionally, the linkage between the amino acid and naphthalene influences the mechanical properties, with 2-(Naphth-2-yloxy)acetic acid providing stiffer gels. Other examples include the use of naphthaleneimide [145], pyrene [128], bipyridine [131], and alkyl chains [129]. Notably, *N*-capped Lauroyl-Phe formed hydrogels (~2000 Pa) comprising flat 2D sheets, while Palmitoyl-Phe formed fibrils that yield a viscous solution [129]. Additionally, while carboxybenzyl-protected amino acids generally fail to form hydrogels, leading to other nanostructures [146], *N*-capping with cinnamoyl can afford gels a slow pH decrease and achieve a G′ ~10^3^ Pa. This makes cinnamoyl the minimum structure motif for *N*-capped phenylalanine to form hydrogels. Garcia et al. [147] also reported hydrogels of Phe *N*-capped with 4-nitrobenzoyl, showing fast gelation, high resistance to applied stress, and thermal reversibility. These gels also enabled high fibroblast and keratinocyte cell viability and displayed mild antimicrobial activity against *E. coli.*

### 3.2. Dipeptides: Chemical Structure of Dipeptide-Based Hydrogels

As with single amino acids, the matrix formation of dipeptide-based hydrogels is closely related to the elongated one-dimensional (1D) supramolecular architectures, such as ribbons or fibers. These assemblies fill the space and form mutual non-covalent entanglements, thereby entrapping water in a three-dimensional (3D) network. Chemically, in dipeptide sequences, the amide bond drives intermolecular hydrogen bonding, which can induce self-assembly. Additional functional groups at the C-terminus, N-terminus, or in the amino acid side chain generally support the formation of non-Newtonian matrices by providing auxiliary chemical points to enlarge the intermolecular force network. The aromatic moieties, in particular, are associated with improved gelation capability, stabilizing favorable conformations for the initial steps of self-assembly. Among all dipeptides, Fmoc-FF represents the paradigm of a dipeptide-based gelator and is, thus, reviewed separately. Other Fmoc-capped dipeptide sequences and differently N-capped diamino acid gelators are also discussed. In the following section, the amino acid sequence in N-capped dipeptides is mentioned with the letter codes for a matter of simplicity.

#### 3.2.1. Uncapped Dipeptides

Although less explored than other gelators, uncapped dipeptides can also form gels, often containing at least one phenylalanine residue, as summarized in Table 2. Kralj et al. [148] studied the effect of heterochirality and halogenation in the self-assembly of Phe-Phe. Unlike L-Phe-Phe, which formed heterogeneous microtubes, the stereoisomer D-Phe-Phe could form stable, thermoreversible hydrogels consisting of homogeneous nanofibrils approximately 4 nm thick, comprising a water channel covered by two layers of peptides. The authors attributed the differences to the stereoconfiguration, which dictates the handedness of the screw-sense from N- to C-terminus. In D-Phe-Phe, this handedness is right-handed, increasing the intramolecular hydrophobic contact area between Phe side chains, reducing interchannel hydrophobic contacts, and consequently reducing fiber bundling. Halogenation was also found to enable the tunability of intra- and intermolecular hydrophobic interactions while preserving the water channels. An intermediate level of fiber bundling was observed compared to L-Phe-Phe and D-Phe-Phe, influencing the mechanical properties. Interestingly, the substitution with the bulky iodine disrupted the water channel assembly, leading instead to the packing into an amphipathic layer. Additionally, no halogen bonding was observed, indicating that iodine only increased hydrophobicity and steric hindrance, stabilizing the interdigitation of Phe zippers.

Conte et al. [149] demonstrated that Phe-Phe and the C-terminal aminated derivative could form metastable hydrogels through a solvent switch method followed by ultrasonication. Sonication led to the formation of nanofibers in addition to the remaining nanotubes, whereas only amorphous aggregates appeared when the solvent switch was done alone. The authors proposed that the sonication may induce a fast change in solubility after the solvent switch, facilitating the formation of numerous nucleation sites, which favors the organization into nanofibers instead of extended 2D β-sheets that are obtained without sonication. Notably, similar to the amino acid derivatives, the C-terminal aminated derivative displayed lower mechanical strength than the unmodified Phe-Phe.

The replacement of phenylalanine with leucine (both L and D isomers) was reported to enable gelation, except for L-Phe-L-Leu [150]. Heterochirality was observed to promote hydrogelation, as D-Phe-L-Leu could form gels, and in the case of Leu-Phe, it led to a decrease in the gelation time. The authors associated this effect with a change in the hydrophobicity arising from the amphipathic conformation in heterochiral peptides, in which the segregation between hydrophilic and hydrophobic regions enables the self-assembly into stable structures. Additionally, the formation of extended networks of hydrogen bonds and Phe zippers was found to be a distinctive feature of stable gels, commonly absent in non-gelling peptides.

**Table 2:** List of unprotected dipeptide hydrogels self-assembly parameters, mechanical properties, melting temperature (T_m_), fibril cross-section, highlights and application. The critical gelation concentration (CGC) and gelator concentration employed in the rheological assays [Gel] are included. The orders (or range) of the limit strain of the linear viscoelastic regime (LVR), critical strain (γ), storage (G′), and loss (G″) moduli are indicated unless the values are detailed in the respective manuscript.

Gelator	Method	Media	pH	CGC (mM)	[Gel] (mM)	G′ (Pa)	G″ (Pa)	LVR (%)	γ (%)	T_m_ (°C)	Fibril (nm)	Highlights	Application	Ref.
L-Phe-Phe	pHE	Buffer	7.3	20	20	22	3.7	10	10	46	200–1000	-	-	[148]
	SE + US	HFIP/ANS	8	8	8	1780	10^2^	-	-	-	~10	-	-	[149]
Phe-ΔPhe	HC	Buffer	7	6.4	6.4	209,000	19,700	-	-	-	15–20	-	Drug delivery	[151]
D-Phe-Phe	pHE	Buffer	7.3	20	20	22.9	1.5	100	100	44	4.3	Thermoreversible	-	[148]
2-F-Phe-Phe	pHE	Buffer	7.3	15	15	8	0.5	10	10	44	11.4	Thermoreversible	-	[148]
3-F-Phe-Phe	pHE	Buffer	7.3	10	10	6.1	0.3	100	100	47	50–500	Thermoreversible	-	[148]
4-F-Phe-Phe	pHE	Buffer	7.3	7	7	20.7	1.2	100	100	42	26.9	Thermoreversible	-	[148]
4-I-Phe-Phe	pHE	Buffer	7.3	4	4	17.7	1.3	100	100	74	63	Thermoreversible	-	[148]
Phe-Phe-NH_2_	SE + US	HFIP/ANS	8	4	4	30,100	10^3^	-	-	-	~10	-	-	[149]
Leu-ΔPhe	US	Buffer	7	15.2	19.1	12,000	10^3^	-	-	-	>100	-	-	[152]
L-Leu-Phe	HC	PBS	7.4	40	40	10^4^	10^3^	10	100	-	-	-	-	[150]
D-Leu-Phe	HC	PBS	7.4	40	40	10^4^	10^3^	10	100	-	12	-	-	[150]
D-Phe-Leu	HC	PBS	7.4	20	20	10^3^	10^2^	-	0.1	-	-	Not stable	-	[150]

CGC: critical gelation concentration; T_m_: gel–sol transition temperature; G′: storage modulus; G″: loss modulus; mM = millimol/L. pHE = pH-exchange (“pH-switch), SE: solvent exchange; US: ultrasound; HC: heating/cooling.

Other uncapped dipeptide-based gelators include the use of α,β-dehydrophenylalanine, in which the conformation constrain in the peptide backbone favors the self-assembly into hydrogels. For instance, Chauhan et al. [151] reported the formation of mechanically strong Phe-ΔPhe gels near physiological pH, displaying a low critical gelation concentration. Vesicle-like structures were observed at the end nodes of fibers and were suggested as structural precursors to the fibril branching. The same group also reported the formation of stable, strong Leu-ΔPhe gels under mild physiological conditions [152].

#### 3.2.2. The Case of Fmoc-FF

**Figure 5:**
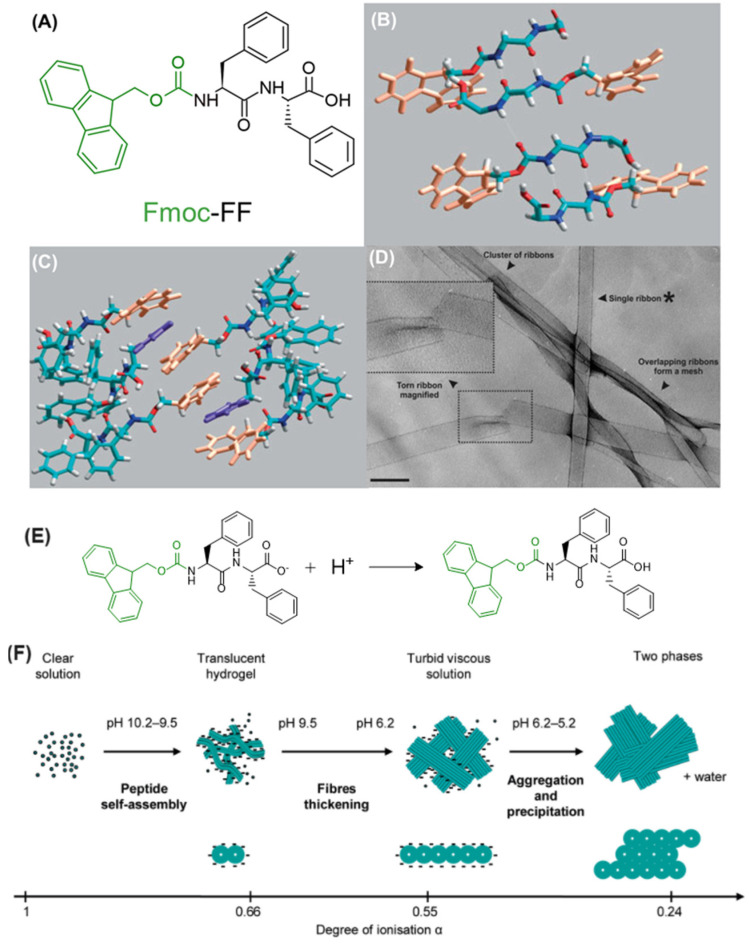
(**A**) Fmoc-FF chemical formula. (**B**) Dipeptide monomers arranged into antiparallel β-sheet. (**C**) Interlocking of Fmoc- chemical groups from alternate β-strands. In the model, Fmoc and the phenyl groups are identified using orange and purple colors, respectively. (**D**) TEM (transmission electron microscopy) microphoto of Fmoc-FF xerogel (scale bar reported is for 500 Å). In the TEM photo, the labeled ribbon was used by the authors for other morphological analyses [167]. The figure is adapted from Ref. [167]. Copyright with permission from Wiley-VCH. (**E**) From high to low pH, the carboxylic acid is protonated, decreasing electrostatic repulsion and, thus, favoring self-assembly. (**F**) Schematic representation of the self-assembly mechanism of Fmoc-FF as a function of the peptide degree of ionization, α. The figure is adapted from Ref. [161].

Diphenylalanine homopeptide (FF), representing the core of Aβ1-40 and Aβ1-42 primary sequences, is a minimal aromatic motif for aggregation in Alzheimer’s β-amyloid [153]. FF can self-assemble through a hierarchical pathway to generate nanotube architecture with a length of ~100 µm, driven by π-π staking and H-bonding interactions [154]. As a consequence of a simple chemical structure, aggregative versatility, and simplicity, diphenylalanine is considered the molecular paradigm for the analysis of phenomena related to peptide self-organization. This prospective reference was reinforced by the possibility of chemical modifications, leading to a library of FF-based analogs [155,156,157,158], including the fluorenylmethoxycarbonyl (Fmoc) *N*-capped sequences (Fmoc-FF, Figure 5A) [159,160,161]. Fmoc-FF is among the most studied ultra-short peptides to develop hydrogels, enabling stable, self-supporting matrices at pH values compatible with physiological applications (e.g., drug delivery, nanogels production, tissue engineering, and optical engineering) [162,163,164]. Fmoc-FF can also serve as a structural component for multicomponent hydrogels [165,166].

Structurally, Fmoc-FF monomers assemble into fibrillary architectures due to favorable molecular stacking, with dimensions and an ultrastructure very similar to amyloid fibrils. The Fmoc-FF multiscale aggregation model, based on circular dichroism (CD) and Fourier-transformed infrared spectroscopies (FT-IR) and supported by scattering gel diffraction studies [167], shows that the monomers initially arrange in an anti-parallel β-sheet secondary structure (Figure 5B) with π-stacking of the Fmoc- groups (Figure 5C). Subsequently, four twisted anti-parallel β-sheets interlock through lateral π-π interactions, forming a nanocylindrical structure characterized by an external diameter of ~3.0 nm. These fibrillar aggregates further self-assemble laterally, generating large flat ribbons (detected using TEM microscopy, Figure 5D), which form macroscopic gels via mutual entanglements.

Fmoc-FF gelation can be induced using three major methodologies: pH exchange, solvent switch, and catalytic methods. These approaches involve changing the initial physicochemical environmental conditions to trigger gelation. Specifically, the pH switch method consists of dissolving the Fmoc-FF in an aqueous solution at elevated pH (pH ~ 10.5), followed by progressively lowering the pH using HCl or glucono-δ-lactone (GdL) [65,167]. At high pH, the peptide’s C-terminal carboxylic acid is deprotonated. As the solution acidifies, protonation occurs, leading to gelation (Figure 5E,F). Fmoc-FF gelation can also be achieved via the solvent switch method. In this approach, the peptide is dissolved by using an organic phase able to produce a high concentration solution (generally 100 mg/mL), and gelation is trigged by adding water. Common solvents for Fmoc-FF dissolution include DMSO and 1,1,1,3,3,3-hexafluoro-2-propanol (HFIP) [168,169]. Lastly, self-aggregation can be induced by the chemical conversion of precursors (unable to self-assemble) into gelling building blocks via catalytic conversion, such as the hydrolysis of charged or steric groups blocking fiber formation [170].

Regardless of the formulation strategy, Fmoc-FF generally forms a reproducible and similar supramolecular structure. The self-assembling conditions and/or pathways constitute the parameters that affect the macroscopic and microscopic architecture, which in turn modifies the rheological response and functional matrix features [171]. For example, more homogeneous matrices are obtained by controlling the pH homogeneity in pH switch procedures. A fast decrease in pH by using HCl can result in local fiber formation and inhomogeneous gels as a consequence of faster gelation kinetics compared to the mixing kinetics. Reproducible and more homogeneous matrices can be formulated by coupling acidification with a heating/cooling cycle [161]. The heating step can dissolve the kinetically trapped aggregates formed during the local acidification, which after cooling can increase the G′ value of Fmoc-FF gels from 1–10 Pa to 10^4^ Pa. Agitation, whether low shear or high shear, can also affect the mechanical response, increasing the G′ value from ~1000 Pa for high shear-prepared gels to ~4000 Pa for low shear-prepared gels [166]. Regarding the organic solvent in the solvent switch method, no differences in mechanical response were reported for Fmoc-FF gels using DMSO or HFIP [172].

#### 3.2.3. Fmoc-Capped Dipeptides

**Figure 6:**
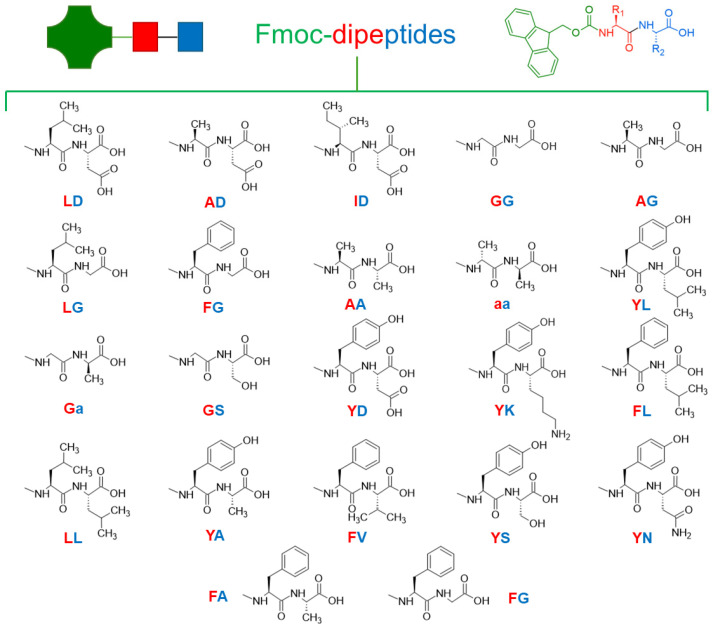
Schematic representation of Fmoc-dipeptides and their chemical formula. Amino acids are identified by using a letter code.

The Fmoc-FF gelation and the use of Fmoc as a standard solid-phase peptide synthesis capping group promoted the study of the self-assembling propensity of other dipeptides (Table 3). The chemical structures of the discussed sequences in this section are collected in Figure 6.

Vergners et al. [173] designed a library of seven Fmoc N-capped dipeptides. Three of these (Fmoc-Leu-Asp, Fmoc-Ala-Asp, and Fmoc-Ile-Asp at 0.5, 6.7, and 0.4 wt%, respectively) could form gels by dissolving the peptide in water at 100 °C, producing gels after cooling down below 60 °C, which displayed a thermoreversible behavior [173]. Rheological analysis showed a G′ of 80 Pa for Fmoc-Leu-Asp (2 mg/mL, ν = 1 rad/s, 60 °C) in line with the macroscopic gel disassembly by mechanical agitation. The other Fmoc-capped peptides (Fmoc-Gly-Gly, Fmoc-Ala-Gly, Fmoc-Leu-Gly, Fmoc-Phe-Gly, Fmoc-Ala-Ala, Fmoc-YL, Fmoc-YA, Fmoc-YS) could also form gels, but the rheological characterization was not completed [160,174].

Synthesized as an intermediate, Fmoc-*aa* (*a* represent Ala in *D*-configuration) inspired the analysis of different Fmoc-capped dipeptides, specifically Fmoc-AA, Fmoc-GG, Fmoc-G*a*, Fmoc-GT, and Fmoc-GS) [175]. Fmoc-*aa* and Fmoc-AA efficiently formed gels around 4 mmol/L, and the substitution of the two alanine residues with glycine (Fmoc-GG) increased the critical gelation concentration (CGC) to 11 mmol/L, while 46 mmol/L and 56 mmol/L were reported for Fmoc-G*a* and Fmoc-GS, respectively. Interestingly, Fmoc-GT failed to gel, possibly as a consequence of a relatively large side chain of threonine. This analysis suggested that an increased steric hindrance can reduce the hydrogel formation ability. Additionally, for Fmoc-*aa*, Fmoc-AA, and Fmoc-GG, a sol–gel transition was detected as a function of pH and temperature, with the pH = 3 found to be optimal for gel formation. The dipeptides precipitated at pH < 3 and dissolved immediately at pH = 6, with the pH responsiveness being completely reversible.

An antiparallel β-sheet hydrogen bonding arrangement was identified as a secondary structure element for the Fmoc-YL amphiphile hydrogelator, supported by Fmoc-interlocked aromatic stacking. An achiral supramolecular organization was detected, and AFM analysis showed that fibers mutually entangle, forming a dense bundle of fibers (40−200 nm in width). Viscoelastic characterization highlighted the soft nature of the gel, with a G′ = 190 Pa [176].

To explore the possibility of hydrogel printing, Fmoc-tyrosine-aspartic acid (Fmoc-YD) and Fmoc-tyrosine-lysine (Fmoc-YK) were synthesized [177]. Both peptides displayed self-assembly behavior, producing transparent and self-supporting hydrogel (10 mM) formed by helical fibrils. Fmoc-YD fibrils had a thickness of ~18 nm and a helical pitch of ~450 nm, while Fmoc-YK fibrils had a thickness of ~5 nm and a helical pitch of ~65 nm. Rheological data suggested that the presence of a positive charge disturbs more than a negative one [177].

A series of Fmoc-capped leucine-containing dipeptides (Fmoc-FL, -YL, -LL) and Fmoc-YA were used to obtain self-healing, shear-thinning hydrogels and studied for their extrusion properties. Interestingly, the rheological response of the four Fmoc-dipeptide hydrogels strongly correlated with the log P value of each sequence. Enhanced hydrophobic interactions in Fmoc-FL correlated with a higher G′ value and immediate self-healing property. Additionally, the Fmoc-FL hydrogel displayed a mixture of nanofibers and straight rods, which contributed to the enhanced mechanical rigidity [178].

The Fmoc-FV sequence could also self-assemble, forming a gel consisting of a fibrous network with a fibril thickness of ~30 nm, in which G′ increases with temperature. The analogs Fmoc-FG and Fmoc-GF formed fibers with 30 nm and 10 nm fibril thickness, respectively, indicating that the isopropyl side chain of valine positively impacted fiber thickness, forming a more rigid matrix [179].

The combination of intermolecular interactions and molecular packing was assumed to explain the higher gel strength of Fmoc-YS compared to Fmoc-YT and the non-gelling Fmoc-YQ. The inability of asparagine-containing sequences to form hydrogels (producing nanosphere architectures) highlighted that the side chain properties and length at position two of the dipeptide sequence can strongly impact the molecular packing. Additionally, the weaker nature of the Fmoc-YT hydrogel further indicated that amino acid order determines the mechanical properties [180].

A significative increase in G′ response was detected in Fmoc-FG, Fmoc-FA, and Fmoc-LG when GdL was used to induce gelation through acidification instead of HCl. In line with other dipeptide-based gels, this suggested that the acidifier impacts the homogeneity, reproducibility, and mechanical strength. The higher density of long fibers formed using GdL was further related to the final mechanical properties and preparation procedure [65].

**Figure 7:**
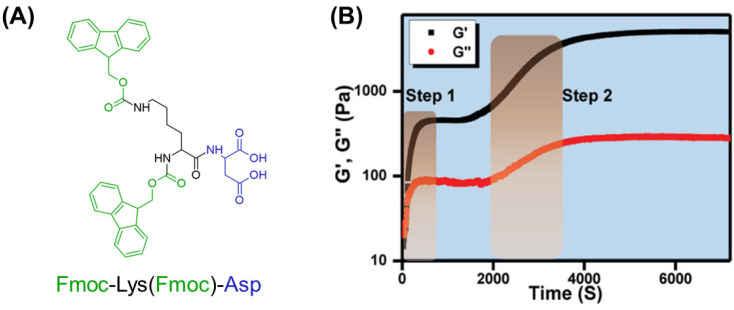
(**A**) Chemical structure of Fmoc-Lys (Fmoc)-Asp. (**B**) Time-dependent G′ and G″ trends exhibit a two-step growth (gel at 0.5 wt%). The figure is adapted from Ref. [181]. Arranged after a copyright required permission from Wiley-VCH.

A minimalistic dipeptide-based low molecular weight gelator, namely Fmoc-Lys (Fmoc)-Asp (Figure 7A), was reported as a molecular starting point for designing a novel Fmoc-based dipeptide. This building block can gel, forming unbranched fibers via a two-step assembly (Figure 7B), at a very low 0.002 wt% concentration. A concentration-related rheological response was also demonstrated [181].

#### 3.2.4. Other N-Capped Dipeptides

Following the success of Fmoc-modified dipeptides, other aromatic chemical entities were explored as N-terminus modifiers to enhance gelation properties. This approach has led to the development of several self-assembling entities. Given their chemical similarity to Fmoc derivatives, these dipeptides are expected to display similar aggregation and gelation features.

**Figure 8:**
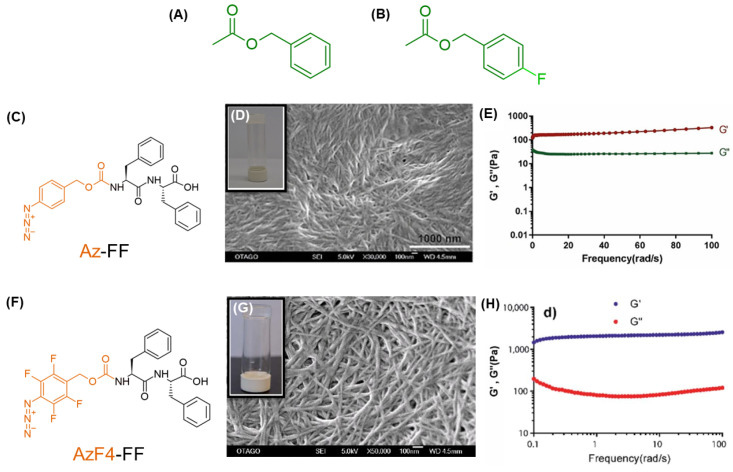
Chemical structure of (**A**) benzyloxycarbonyl- and (**B**) 4-F-benzyloxycarbonyl capping groups. Az-FF (**C**,**F**) AzF4-FF peptide structures. SEM images for a hydrogel of (**D**) Az-FF (scale bar of 1000 nm) and (**G**) AzF4-FF with their macroscopical samples in the insert. Panels (**E**,**H**) report the frequency sweep experiment for the matrices formed by Az- and AzF4-FF, respectively. The figure is adapted from Refs. [185,186]. Copyright with permission from Wiley-VCH and from RSC.

One such moiety, benzyloxycarbonyl-(Z-, Figure 8A), is a simple aromatic group that can replace the Fmoc group in solid-phase peptide synthesis (SPPS) and that can be employed to obtain gelators. For example, Z-FF was found to produce hydrogels at 0.2 wt% [182]. Rheological characterization of these hydrogels, prepared at concentrations of 0.5 and 1.0 wt% after a solvent switch (HFIP/H_2_O or acetic acid/H_2_O), revealed no significant differences in gelation based on the solvent used. At a concentration of 1.0 wt%, the storage modulus (G′) was higher than 10^5^ Pa, while at 0.5 wt%, it was lower, indicating a dependence on peptide concentration. This finding could be correlated with the contact number between fibers forming the matrices. At 4.8 mM, Z-FF formed opaque gels with a critical gel concentration (CGC) of 0.14 wt%. The gel exhibited a G′ of 2000 Pa when prepared at 25 °C, though this value decreased to 300 Pa following a heating/cooling cycle from 50 °C [129]. Additionally, a library of dehydrodipeptides Z-L-Xaa-*Z*-ΔPhe-OH (Xaa = Met, Phe, Tyr, Ala, Gly) was also synthesized and screened for hydrogel formation by Veloso et al. [183]. Derivatives with Xaa = Met, Phe, and Tyr were found to form hydrogels through pH exchange by GdL. Importantly, at a concentration of 0.3 wt%, the gels based on the dehydropeptide with Xaa = Phe (G′ ~100 kPa) were 100× and 10,000× stronger than the peptides with Met and Tyr, respectively. Additionally, the gels were frequency-independent, and the dehydropeptide with Xaa = Phe could also form gels through a heating/cooling cycle in phosphate buffer (0.1 M, pH = 7.3).

Derivatives containing diphenylalanine *N*-capped with lauric (C12-FF), myristic (C14-FF), or palmitic acid (C16-FF) were also reported. Among these compounds, only C16-FF demonstrated the ability to form a gel at a concentration of 0.1 wt% with a storage modulus (G′) of 300 Pa [129]. The gelation was studied through the addition of either calcium or magnesium nitrate salts [61]. At pH = 11.7 and a concentration of 0.5 wt%, viscosity values of 44, 17 and 509 η/mPa s^−1^ for C12-, C14-, and C16-FF, respectively, were reported. Turbid gels were formed only with C14-FF (G′ = 3400 Pa, G″ = 732 Pa) and C16-FF (G′ = 2361 Pa, G″ = 334 Pa).

Further studies of the gelation propensity involved fluorinated benzyloxycarbonyl- (Z-) analogs, specifically benzyl-FF (Bz-FF) and its 4-fluorobenzyl-FF (Figure 8B, BzF4-FF) related analog. [184]. BzF4-FF formed fibrillary architectures and self-supporting hydrogels in the range of 1.5 wt% to 5.0 wt%, while Bz-FF resulted in a viscous solution with simultaneous precipitate formation. Rheological studies demonstrated that only the fluorinated analog formed a gel, with a G′ value exceeding 5700 Pa. Geometry optimization revealed that the gelation propensity was influenced by the “sandwich” configuration of BzF4-FF compared to the T-shaped configuration of Bz-FF.

An N-4-azidobenzylcarbamate-FF (Az-FF, Figure 8C) was proposed as a building block for stimuli-responsive matrices for drug delivery applications due to its ability to induce a gel–sol transition through radical modification of the non-covalent interactions [185]. Az-FF could form gels at 0.5 wt% through the solvent-switch method with DMSO and water or buffers (PBS or acetate). Transmission electron microscopy (TEM) and scanning electron microscopy (SEM) analyses showed the formation of highly dense and thin fibers (SEM, Figure 8D). Despite this, Az-FF gels exhibited a low storage modulus (200 Pa) as measured by frequency sweep experiments (Figure 8E). The authors explored a trans-cyclooctene (TCO) triggered hydrogel by substituting the N-4-azidobenzylcarbamate group with 4-azido-2,3,5,6-tetrafluorobenzyl carbamate (AzF4-FF, Figure 8F). Synthesized similarly to Az-FF, AzF4-FF formed hydrogels with elongated fibrillar aggregates (Figure 8G) with a lower CGC of 0.1 wt%. Additionally, it displayed an improved rigidity (G′ = 1500 Pa, Figure 8H) compared to the Az-FF derivative, even at lower concentrations [186].

*N*-protected cinnamoyl diphenylalanine dipeptide (Cin-FF) was found to self-assemble into helical fibers, forming a self-supporting semi-transparent matrix through a thermally triggered gelation followed by sonication [187]. The assembly consisted of elongated flat ribbons, approximately ≈50 nm wide, which interconnected into broader fibers (~144 nm) that then fold to form helical structures. The major (two-fold) and minor (one-fold) helix turns were ~1.2 μm and ~870 nm, respectively, with a G′ value of ~226 Pa. Compared to Fmoc-FF (G′ ~9500 Pa), it was noted that the preparation method significantly influenced the mechanical response, resulting in different stiffnesses despite similar elasticity and comparable G′-G″ cross points in their amplitude sweep profiles.

The FF sequence was also modified at N-terminus with indolacetic acid (In-FF), resulting in the formation of architecture rich in β-sheet secondary structuration. In-FF gels can be prepared using several approaches, such as dissolution at 90 °C in PBS, pH switching, or DMSO solvent switching. When prepared using the pH switch method, these gels exhibited extremely high G′ values. This high G′ was associated with additional hydrogen bonding from the indole moiety, π-π interactions between the indole and phenylalanine residues, and the formation of large fibrous networks visible to the naked eye. TEM micrographs revealed that fiber thickness ranged from 100 to 400 nm, with isolated nanofibers approximately 2 nm in height. Notably, no fiber branching was observed in either TEM or AFM images; instead, it displayed a high degree of fiber bundling. As with Fmoc-FF, the G′ values for In-FF gels depended on the preparation method: pH switch method gels had G′ values around 3 × 10^5^ Pa, whereas gels prepared by solvent switch or temperature methods had G′ values around or below 10^4^ Pa [188].

**Figure 9:**
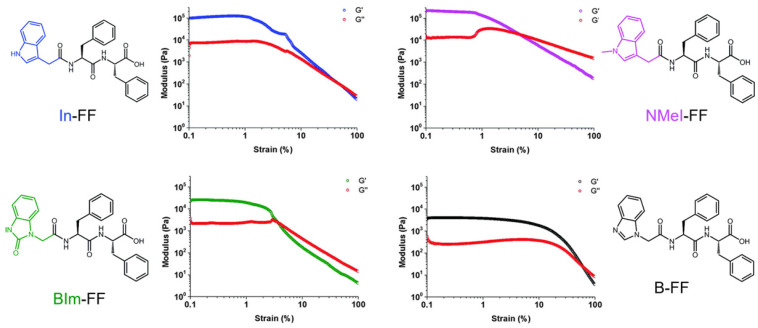
Rheological characterization and chemical structure of indole-FF (In-FF), N-methylindole-FF (NMeI-FF), benzimidazolone-FF (BIm-FF), and benzimidazole-FF (B-FF) derivatives. The figure is adapted from Ref. [189]. Copyright with permission from RSC.

A comparative study about strictly related building blocks of FF N-capped via indole heterocyclies, (indole-FF (In-FF), N-methylindole-FF (NMeI-FF), benzimidazolone-FF (BIm-FF), and benzimidazole-FF (B-FF)) highlighted that mechanical properties are strongly influenced by the nitrogen substitution in the heterocyclic capping group (Figure 9) [189]. All peptides formed hydrogels through pH switch using GdL, with ultimate gel pH values around 4–5. Interestingly, the clog *p* values of the peptides correlated with the respective CGC. Circular dichroism studies suggested that the methylated indolic nitrogen in NMeI-FF interfered with β-sheet formation (absent in In-FF), underscoring the role of the capping group in the structural properties. In-FF and NMeI-FF displayed a similar linear viscoelastic region (LVR) up to a strain of 1% and showed reversibility. In contrast, the hydrogels based on BIm-FF and B-FF displayed LVRs extending to 3% and 10%, respectively. The peptides with a larger degree of nitrogen substitution in their capping group led to softer hydrogels and greater strain tolerance. The crossover point value varies from 60% for B-FF to as low as 3% for BIm-FF. The storage moduli of the indole-based hydrogels, In-FF and NMeI-FF, exceeded 10^5^ Pa, indicating a significant stiffness. Despite the previously reported rigidity of In-FF hydrogels, the high storage modulus of the NMeI-FF derivative was unexpected. This suggested that mechanical properties are influenced less by hydrogen bonding and more by the degree of nitrogen substitution. For instance, BIm-FF and B-FF, though displaying similar stiffness, had an order of magnitude lower stiffness compared to indole-based gels, further highlighting the impact of nitrogen substitution on the heterocyclic capping group.

**Figure 10:**
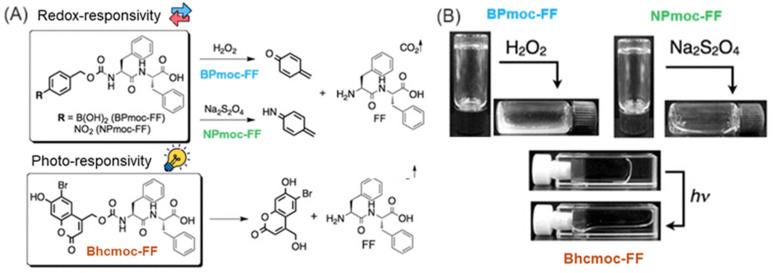
(**A**) Chemical structure and stimuli-responsive degradation mechanisms of BPmoc-FF (blue), NPmoc-FF (green), and Bhcmoc-FF (orange) matrices, and the respective (**B**) gel-sol transitions. The figure is adapted from Ref. [195]. Copyright with permission from Wiley.

Carbazole-capped (Figure 10A) diphenylalanine (CBz-FF) was found to self-assemble into hydrogels via a pH switch, forming a β-sheet secondary structure [190]. CBz-FF is noted as a “supergelator”, with a critical gelation concentration of 0.03 *w*/*v*%. This low gelation concentration is attributed to the carbazole group, which allows CBz-FF to gel at concentrations where Fmoc-derivatives cannot. The carbazole group favors the formation of smaller molecular fibers, averaging 1.7 ± 0.3 nm in diameter, compared to the 2 nm fibers of Fmoc analogs. Despite the comparable fiber size, their morphologies differ significantly: CBz-FF forms a highly branched network of nanofibrils that are disjointed rather than continuous. At 1.0 wt%, CBz-FF hydrogels displayed a storage modulus (G′) of 0.5–0.7 kPa.

Aldilla et al. [191] introduced a new class of short peptides with a glyoxylamide capping group (5X-GL). The glyoxylamide group includes two carbonyl groups oriented in different spatial directions, enhancing hydrogen bonding interactions. Various derivatives could form gels through different methods, such as solvent switch, heating/cooling, and pH switch. However, 5-H-glyoxylamide-FF (5H-GL-FF) was unique in forming hydrogels through a slow pH decrease with GdL, producing a softer hydrogel (G′ ~103 Pa) compared to the other derivatives (5F, 5CH_3,_ and 5Br). These results were correlated with the LogP values, emphasizing the importance of the hydrophobic/hydrophilic balance. Notably, 5F, 5CH_3_, and 5Br derivatives could produce organogels in toluene and isopropyl alcohol.

A pyrene (Pyr)-YL amphiphile hydrogelator was compared with its Fmoc-protected analog, revealing that the increased aromaticity volume of pyrene contributed to the superior mechanical properties and recovery of Pyr-YL (G′ = 390 Pa) compared to the Fmoc analog (190 Pa) [176].

ThNap-FF building block, *N*-capped with a tetrahydronaphthalene (ThNap), could form gels upon salt addition, forming a rigid matrix (G′ ~5.5 × 10^4^ Pa) and demonstrating recovery from shear stress (66% over 5 min). An analog sequence, ThNap-VG, did not gel, indicating a sequence-dependent behavior [61]. When prepared using a DMSO/H2O (80/20, *v*/*v*) solvent switch, ThNap-FF produced more homogeneous and reproducible gels. This approach enabled the creation of gel noodles with tunable mechanical responses (from 5 to 20 kPa) depending on the production flow rate [13].

The conjugation of nucleobases, specifically thymine, adenine, cytosine, and guanine, was explored to design supramolecular nanofibers and hydrogels. Rheological characterization at 2.0 wt% indicated that purine bases generate stronger matrices, and the differences were associated with the pKa nucleobases [192].

A 7-hydroxycoumarin-capped diphenylalanine (Cou-FF) formed gels through a pH switch with GdL, comprising a fibril network with a storage modulus of 82 kPa [193].

Ferrocene-FF (Fc-FF) was observed to undergo a conversion from nanospheres to nanofibers under shaking, forming a yellow self-supporting hydrogel with a G′ of 1000 Pa. Although the mechanical response was lower compared to Fmoc-FF, the ferrocene group was proposed as a molecular kinetical determinant of aggregation.

Novel UV-Vis-responsible peptide-based hydrogels were developed by modifying different dipeptide sequences with the azobenzene (Azo-) moiety [194]. The Azo group, consisting of two phenyl rings linked by an N=N double bond, facilitated the self-aggregation of Azo-FF through intermolecular π-π stacking. Upon light irradiation at λ = 365 nm, Azo groups reversibly switch between *E*- and *Z*- conformations, altering aromatic interactions and affecting gelation. The library of peptides included several dipeptide sequences (Phe-Glu, Gln-Tyr, Glu-Phe, Gln-Gln, Phe-Ser, Gln-Ala, Ser-Phe, Glu-Ala, Phe-Ala, Arg-Ala, Ala-Phe, Arg-Phe, Phe-Phe, Arg-Gln, Phe-Tyr, Ser-Ala, Tyr-Ala, Lys-Ala, Ala-Tyr, Arg-Lys, Tyr-Tyr, Glu-Lys, Glu-Tyr, and Tyr-Lys) that were evaluated for hydrogelation ability. Charged amino acids were found to inhibit gelation, while aromatic residues supported hydrogel formation. However, comprehensive rheological characterization was not carried out.

BPmoc (borono-phenylmethoxycarbonyl), NPmoc (p-nitro-phenylmethoxycarbonyl), and Bhcmoc (6-bromo-7-hydroxycoumarin-4-ylmethoxycarbonyl) derivatives were also used as Fmoc- alternatives for diphenylalanine capping (Figure 10). These compounds formed self-supporting gels with redox, thermal, and pH-responsiveness. Although rheological data were not reported, peptides capped with the more hydrophobic NPmoc and Bhcmoc groups exhibited low critical gelation concentrations of 0.35 and 0.40 wt%, respectively, highlighting a significant role of the capping group in gel formation [195].

Similarly, to Fmoc-FF, 6-nitroveratryloxycarbonyl-diphenylalanine (Nvoc-FF) generates a 3D, self-supporting, nanofibrous hydrogel using a DMSO/water solvent switch approach. Additionally, the Nvoc-FF hydrogel exhibited good mechanical properties with a G′ value of 40 kPa and a gel–sol transition under UV-Vis exposure at 365 nm [45].

To create more biocompatible molecular hydrogelators, Xu et al. [196] developed a class of hydrogelators based on (naphthalen-2-yloxy)acetic acid, naphthalen-, and methyl-naphthalen-conjugated dipeptides (GG, GA, G*a*, GS, GV, and GL sequences). Only the (naphthalen-2-yloxy)acetic acid derivatives were able to form gels, suggesting that the -OCH_2_- spacer can promote the correct conformations in the molecular entities and act as a positive hydrogen bond acceptor. Excellent hydrogelation abilities were noted for Nap-Ga and Nap-GaA, with a CGC of 0.07 wt% and a gel–sol transition around 50 °C. Transmission electron micrographs (TEMs) revealed uniform helical nanofibers in both gels, with a diameter of ~30 nm and pitches near 60 nm. Comparing the gelation abilities of GG, G*a*, and GA sequences, the alanine methyl group decreased the backbone flexibility and, in turn, the conformational entropy. For Nap-GS, the steric hindrance induced by the hydroxylmethyl group affected the intermolecular interactions, leading to less stable hydrogels. The role of steric hindrance is further highlighted by the relatively large and hydrophobic side chains of valine and leucine in the GV and GL derivatives, which did not afford gels. Thus, increased steric hindrance and total hydrophobicity can reduce the gelation tendency.

**Figure 11:**
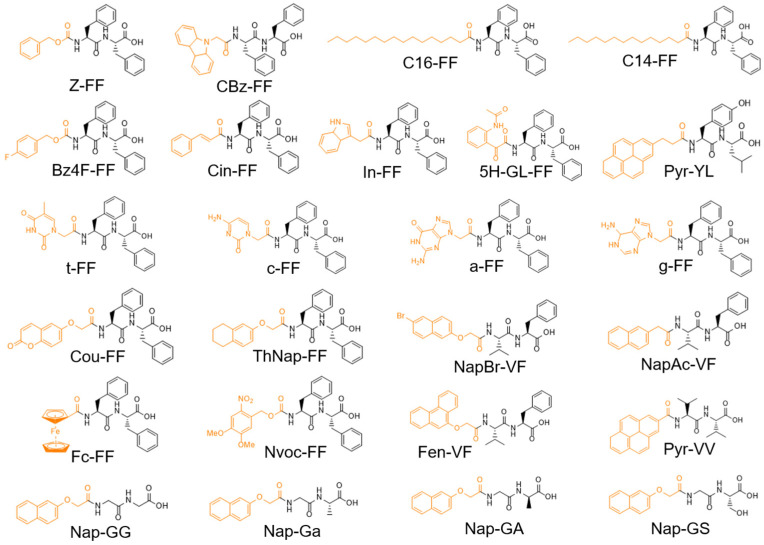
Chemical structure of the discussed dipeptides *N*-capped with different groups.

Using a combinatory approach, a library of 34 dipeptides (sequences Val-Val, Val-Ala, Val-Gly, and Val-Phe) N-capped with different aromatic groups (2-(naphthalen-2-yloxy), 2-(naphthalen-1-yl)acetamido, 2-(phenanthren-9-yloxy), 2-(anthracene-9-carboxamido), and their Br or diBr substituted analogs) was studied [197]. As mentioned, the self-assembly is strongly affected by the preparation method (pH- or solvent-triggered approach). A larger number of the dipeptides are known to assemble into a fibrillar network through a pH-triggered approach rather than the solvent switch. From this expanded library, it was found that dipeptides containing at least one phenylalanine are significantly more likely to form gels. The likelihood of gelation increased when the naphthalene ring was replaced with phenanthrol. All dipeptides N-capped with carbazole formed gels through the pH-triggered method, while anthracene-capped dipeptides could not form gels. Some examples from the library are included in Table 4 and represented in Figure 11.

**Table 3:** List of Fmoc-dipeptide hydrogels self-assembly parameters, mechanical properties, melting temperature (T_m_), fibril cross-section, highlights and application. The critical gelation concentration (CGC) and gelator concentration employed in the rheological assays [Gel] are included. The orders (or range) of the limit strain of the linear viscoelastic regime (LVR), critical strain (γ), storage (G′), and loss (G″) moduli are indicated unless the values are detailed in the respective manuscript.

Gelator	Method	Media	pH	CGC (mM)	[Gel] (mM)	G′ (Pa)	G″ (Pa)	LVR (%)	γ (%)	T_m_ (°C)	Fibril (nm)	Highlights	Application	Ref.
Fmoc-AA	pHE	Water	<4	1.6–16.9	-	-	-	-	-	-	68 ±1 8	-	-	[160]
Fmoc-AD	HC	PBS	7.4	142.3	10	-	-	-	-	-	-	-	-	[173]
Fmoc-AG	pHE	Water	<4	4.3-17.9	-	-	-	-	-	-	30 ± 6 [d]	-	Cell growth	[160]
Fmoc-FA	GdL	Water	3.75	-	14.6	95,600	-	-	-	-	-	-	-	[65]
Fmoc-FG	pHE	Water	<4	4.0–17.8	-	-	-	-	-	-	25 ± 6 [d]	-	Cell growth	[160]
Fmoc-FG	GdL	Water	3.75	-	14.6	41,000	-	-	-	-	-	-	-	[65]
Fmoc-FL	D	PBS	~8	-	20	11,500	>1100	<2	~6		100–150	Fiber and straight rods	Fillers	[178]
Fmoc-FV	pHE	Water	7.4	-	20	800	650	>100	-	-	30 [t]	-	3D cell culture	[179]
Fmoc-GG	pHE	Water	<4	4.2–16.9	-	-	-	-	-	-	33 ± 8 [d]	-	Cell growth	[160]
Fmoc-ID	HC	PBS	7.4	8.5	10	-	-	-	-	-	-	-	-	[173]
Fmoc-K(Fmoc)-D	SE	Water/DMSO		0.03	7	-	-	<100	-	-	-	Conductive gel	DNA binding	[181]
Fmoc-LD	HC	PBS	7.4	10.7	10	80	~15	<0.3	>10	-	-	-	Drug delivery	[173]
Fmoc-LG	pHE	Water	<4	8.5–17.8	-	-	-	-	-	-	22 ± 5 [d]	-	Cell growth	[160]
	pHE − HCl	Water	3.75	-	14.6	5900	-	-	-	-	-	-	-	[65]
	GdL	Water	3.75	-	14.6	184,000	-	-	-	-	-	-	-	[65]
Fmoc-LL	D	PBS	~8	-	20	1500	~300	<1	~10	-	20–50	-	Fillers	[178]
Fmoc-YA	D	PBS	~8	-	20	800	~300	<0.5	~5	-	20–50	-	Fillers	[178]
Fmoc-YD	D	Water	-	-	10	~4500	~2000	<100	~50	-	18 [t]	Helical fibrils	3D Bioprinting	[177]
Fmoc-YK	D	Water	-	-	10	20	8	<2	~30	-	5 [t]	Helical fibrils	3D Bioprinting	[177]
Fmoc-YL	pHE	Water	~7.3	-	10	~390	~190	<0.1	-	-	40–200	Stable ν = 0.1–15.8 Hz		[176]
	D	PBS	~8	-	20	6000	~1000	<2	~10	-	-	Shear-thinning	Fillers	[178]
Fmoc-YN	Enz/pHE	PBS	8	-	10	3010	949	-	-	-	-	-	-	[180]
Fmoc-YS	Enz/pHE	PBS	8	-	10	3400	100	-	-	-	-	-	-	[180]

CGC: critical gelation concentration; T_m_: gel–sol transition temperature; G′: storage modulus; G″: loss modulus. Amino acids are reported as one letter code. mM = millimol/L; ν = frequency; pHE= pH-exchange (“pH-switch); D = dissolution; [t] = thickness; [d] = diameter; [w] = width; [p] = pitch; Enz = enzymatic deprotection; PB: phosphate buffer; DMSO: dimethyl sulfoxide; SE: solvent exchange; US: ultrasound; HC: heating/cooling; GdL: glucono-δ-lactone. AA: acetic acid; Fiber dd = relationship between fiber dimension and concentrations.

## 4. Perspective on the Structure-Property Relationship

The mechanical properties of hydrogels can be affected by different parameters, including the network morphology, stiffness of the network chains, and cross-linking density [199,200]. Adding to the complexity, different networks may exhibit similar scaling behavior with concentration [88,89]. Regarding morphology, the amino acid and dipeptide-based gels commonly display an entangled fibrillar network, which can also include helical fibers, rigid rods, bundles, nanotubes, 2D sheets, stringed nanoparticles, and tape-like structures. However, many studies lack detailed characterization of these gels, such as strain sweep data or specific rheological conditions, complicating the understanding of how morphology affects mechanical properties. The inherent complexity of peptide-based gels and lack of experimental detail makes it difficult to understand the relationship between morphology and mechanical properties. However, despite these challenges, the influence of some parameters can be understood from the more in-depth studies found in the literature.

### 4.1. Influence of the Self-Assembly Pathway

Considering that LMWG-based gels commonly consist of kinetically trapped or metastable states, the self-assembly conditions can strongly impact the resulting properties. The self-assembly conditions include the stimulus magnitude, charge screening, homogeneity, and aging. For example, using GdL instead of HCl can improve gel homogeneity and mechanical properties due to a slower, more uniform pH decrease [64,65,66,67,68]. A larger concentration of enzymes in enzyme-triggered gels [70,71,72,73], or ionic force [60,62,74,75,76], commonly lead to a faster gelation and larger G′ values. The incremental Ca^2+^ content and cation valence as follows: M^+^ < M^2+^ (*s* block) < M^3+^ (*d*, *p*, *f* block) < M^2+^ (*d* block) were also demonstrated to increase the storage modulus [62]. This trend aligns with the Irving–Williams series for ionic binding affinity, as well as with the greater coordination and covalent character achieved with d-block ions. Other parameters that can influence the mechanical properties include the concentration and pH [77]. A larger concentration of peptide leads to more fibers available to entangle/crosslink and a lower pH in N-terminal protected peptides leads to a larger storage modulus [127]**.**

In addition to the mentioned conditions, the self-assembly pathway can influence the morphology of the gel and resulting properties. This is a well-known concept in other supramolecular materials but less explored peptide-based gels. For instance, a slow pH decrease typically leads to a network with more uniform fibers, whereas a rapid pH decrease with HCl results in inhomogeneities [64,65,66,67,68]. Regarding the solvent switch method, peptides often assemble in spherulitic domains of fibers [201,202]. Under strain, the connections between these spherulites can break, but the spherulitic domains remain unaffected and can reassemble after the strain is removed. Conversely, gels prepared with GdL tend not to recover well, potentially due to the fibers being too hydrophobic, which prevents the re-entanglement after removal of the strain. Based on these differences, Draper et al. [10] suggested that gel recovery depends more on fiber distribution than on specific gelator or solvent conditions. However, the literature here summarized in tables reveals some key insights: (1) thixotropic gels can be obtained through both slow pH decrease and solvent switch methods, as well as a heating/cooling cycle; (2) different assemblies, including spherulitic domains, helical fibers, and networks of fibers and tapes, can form thixotropic gels; (3) the apparent assembly pKa and final pH are crucial in determining the recovery capability of the gels. Regarding the latter point, recently, an injectable and reversible gel was reported [43], which could be prepared by dispersing the peptide in a basic solution followed by pH exchange with a buffer solution at physiological pH. Additionally, the Fmoc-Tyr (3NO_2_) could form reversible gels through a heating/cooling cycle near neutral pH, except at more acidic conditions (pH = 5) [122], while Fmoc-Dap (Fmoc) could provide thixotropic gels in a wider pH range (4.9 to 9.1) [127]. Therefore, instead of only the fiber distribution, the revised literature suggests that a specific gelator and solvent, and associated peptide–peptide, solvent–solvent, and peptide–solvent interactions, can determine the range of conditions in which the gel is reversible, as well as the associated morphology and properties. In this way, a peptide such as Fmoc-Tyr (3NO_2_) can provide reversible gels if the gelation is carried near the apparent assembly pKa, but lowering the pH further may turn it too hydrophobic and hinder the structure recovery after strain. Understanding the impact of structural parameters on assembly, morphology, and resultant properties is crucial. However, many studies have only evaluated the frequency-dependence of G′ and G″ values, which is insufficient. Gels developed by different methods can show similar G′ and G″ values and frequency independence but differ significantly in strain sweeps. Therefore, it is important to conduct a comprehensive rheological characterization of the gels for a complete understanding.

### 4.2. Influence of the Chemical Structure

Aromaticity and hydrophobicity are two chemical concepts often recalled for the design of gelling sequences or to improve the mechanical response of gels. Balancing these parameters is essential to prevent precipitation and enable self-assembly. Notably, phenylalanine, the smallest gelling unit, plays a crucial role in the self-assembly into anisotropic structures, as the presence of at least one phenylalanine residue increases the likelihood of forming a hydrogel. Hydrophobic peptides generally lead to greater mechanical rigidity and lower critical gelation concentrations [178], possibly by promoting peptide participation in fiber formation. To modulate hydrophobicity and rigidity, several strategies can be employed. For example, (charged or uncharged) residues can alter hydrophobicity but often limit gel formation conditions, as evidenced by the rare examples of gels based on dipeptides with such residues. Another possibility includes the modification of the aromatic rings with electron-withdrawing or donor groups. However, this modification implies the steric effect and electronic character of the aromatic substituents, which can also strongly influence the resulting properties. For instance, using electron-withdrawing substituent groups has produced thixotropic and/or stronger amino acid-based gels compared to gelators with non-substituted phenylalanine, while electron-donating groups typically improve rigidity more effectively. Halogen substituents on dipeptides could also influence the mechanical properties by adjusting intra- and intermolecular hydrophobic interactions while preserving water channels [148]. However, excessive hydrophobicity or steric hindrance, such as with bulky iodine substituents, can disrupt water channel formation and alter gel properties, though gels may still be achievable. It is noteworthy that, despite the benefit of increasing the steric hindrance, total hydrophobicity, and aromaticity, if not balanced, these parameters can also reduce the gelation tendency. Heterochirality can also promote hydrogelation by creating amphipathic conformations with segregated hydrophilic and hydrophobic regions. Increasing the intramolecular hydrophobic contact area between phenylalanine side chains in heterochiral dipeptides was found to reduce the interchannel hydrophobic contacts and, consequently, diminish fiber bundling [148,150]. These findings were also reported for N-capped dipeptides [196], highlighting the importance of steric effects and aromatic–aromatic interactions.

The incorporation of an N-capping group increases the complexity of the parameters that affect the mechanical properties. For instance, additional parameters include the volume of the capping group, the degree of nitrogen substitution in the case of a heterocyclic capping group, the pKa of the nucleobases for nucleic acid base-protected dipeptides, and the flexibility of the peptide backbone. For instance, a larger aromatic volume in the N-capping group generally leads to more robust mechanical properties and self-healing capabilities. Overall, the peptide structure requires a polarity balance, as replacing the carboxylic acid groups with esters eliminates the gelation capability. Moreover, the presence of amines (positive charge) is known to disrupt gel formation or lead to unstable gels with a weaker storage modulus compared to their counterparts with carboxylic acid. The backbone flexibility also plays a critical role, which generally leads to gels with a larger critical gelation concentration and weaker mechanical rigidity. Thus, decreasing the flexibility by including conformation constraints, such as α,β-dehydroamino acids, was found to favor the self-assembly into hydrogels, resulting in mechanically strong gels near physiological pH and with a low critical gelation concentration [151,152,203,204]. The N-capping with a more conformationally restrained aromatic group, such as 1-naphthaloyl, was reported to form stronger gels than dehydropeptides N-capped with 2-naphthylacetyl, though the latter were prepared at a higher pH [205], which can affect the properties. Instead, larger conformation flexibility in the capping group spacer, such as the -OCH_2_- in (naphthalen-2-yloxy)acetic acid derivatives, have commonly resulted in improved self-assembly and stronger gels [116,196].

## 5. Conclusions and Challenges

The development of materials for innovative applications and the comprehension of their functional performances is strictly related to their self-assembling process. This review shows that unprotected and single protected amino acids, Fmoc- and other differently capped peptides can serve as chemical entities for the development of hydrogels. Distinguished by a multiscale aggregation and self-supporting features, these systems were analyzed from the rheological point of view, looking for a connection between chemistry, sequence, and mechanical response. Regarding the influence of different self-assembly pathways, cavitation rheology is anticipated as a key method to improve the understanding of peptide gels, including the effect of confinement and the vial in which the gel is prepared, as well as the study of gradients and layer-by-layer structures, which are crucial for 3D printing applications. However, more studies are required to fully understand this relationship.

Despite the mentioned advancements, challenges remain in fully understanding the impact of the different structural parameters. Several works do not report essential data such as the mechanical properties of gels, the conditions in which the gels were prepared or measured, detailed mechanical characterization (LVR region and strain sweeps), pH, or critical gelation concentration data. Additionally, some studies compare peptide libraries under different conditions. In addition to the variable preparation methods and concentrations, these pitfalls challenge a consistent understanding of the influence of structural parameters on peptide-based gels and the self-assembly phenomena. Thus, the design of new gelators with the desired properties remains complex. However, it is clear that a general effective structure to obtain gels consists of phenylalanine N-capped with a bulky aromatic group, which can be further modified at the C-terminus with other AA residues. The mechanical rigidity can be easily modified with the halogenation of aromatic groups, aromatic N-capping groups, heterochirality and dehydroamino acids. Furthermore, exploring various self-assembly pathways and conditions is crucial to achieving the desired gel properties. The attention to these points in future works may facilitate a comprehensive analysis of the peptide-based gels material class, opening a venue to a possible predictive mathematical model.

## Figures and Tables

**Table 1:** List of amino acid-based hydrogels self-assembly parameters, mechanical properties, melting temperature (T_m_), fibril cross-section, highlights and application. The critical gelation concentration (CGC) and gelator concentration employed in the rheological assays [Gel] are included. The orders (or range) of the limit strain of the linear viscoelastic regime (LVR), critical strain (γ), storage (G′), and loss (G″) moduli are indicated unless the values were not detailed in the respective manuscript.

Gelator	Method	Media	pH	CGC (mM)	[Gel] (mM)	G′ (Pa)	G″ (Pa)	LVR (%)	γ (%)	T_m_ (°C)	Fibril (nm)	Highlights	Application	Ref.
D-Phe	HC	PBS	-	184	300	-	-	-	-	-	-	-	-	[102]
L-Phe	HC	PBS	-	99	300	-	-	-	-	-	-	-	-	[102]
	HC	Water	6.45	-	303	~200,000	~45,000	-	-	323.6–326.6	437	-	-	[104]
Fmoc-Phe	HC	PB	7.4	2.58	-	~10^3^	~10^2^	-	-	38	4.54–7.24	Tuneable T_m_	-	[113,114]
	GdL	Water	6.1	9.68	25.8	~10^4^	~10^2^	1	10	-	-	-	-	[115]
	GdL	Water	6.6	7.74	25.8	50,199	~2000	0.1	0.2	55	-	Stringed nanoparticles	-	[116]
	SE	Water/DMSO (98:2 *v*/*v*%)	-	-	4.9	39	5	-	-	-	295	-	-	[95,117]
Fmoc-Phe-DAP	NaCl + HC	Water	-	20	33.7	383	59	1	1	-	500–1000	Thixotropic fibrils and nanotubes	Drug delivery	[119]
Fmoc-4-NO_2_-Phe	SE	Water/DMSO (98:2 *v*/*v*%)	-	-	4.9	410	66	-	-	-	12	-	-	[95]
Fmoc-4-CN-Phe	SE	Water/DMSO (98:2 *v*/*v*%)	-	-	4.9	140	17	-	-	-	25	-	-	[95]
Fmoc-4-F-Phe	SE	Water/DMSO (98:2 *v*/*v*%)	-	-	4.9	102	9	-	-	-	26	-	-	[95]
Fmoc-4-NH_2_-Phe	SE	Water/DMSO (98:2 *v*/*v*%)	-	-	4.9	527	61	-	-	-	11	-	-	[95]
Fmoc-4-CH_3_-Phe	SE	Water/DMSO (98:2 *v*/*v*%)	-	-	4.9	280	53	-	-	-	21	-	-	[95]
Fmoc-3-F-Phe	GdL	Water	-	-	7.5	3918	296	-	-	-	126	-	-	[117]
	GdL	Water	-	-	5–15	10^2^–10^3^	10^1^–10^2^	-	-	-	11–19	Thixotropic	-	[118]
	SE	Water/DMSO (98:2 *v*/*v*%)	-	-	5	10^3^–10^4^	~10^4^	-	-	-	22	Thixotropic	-	[118]
Fmoc-3F-Phe-DAP	NaCl + HC	Water	-	20	33.7	21,311	3973	0.1	1	-	20–30	Thixotropic fibrils and tapes	Drug delivery	[119]
Fmoc-F_5_-Phe	GdL	Water	-	-	7.5	4786	449	-	-	-	13	-	-	[117]
	GdL	Water	-	-	5–15	10^2^–10^3^	10^1^–10^2^	-	-	-	24–16	Thixotropic	-	[118]
	SE	Water/DMSO (98:2 *v*/*v*%)	-	-	5	10^3^	10^2^	-	-	-	15	Thixotropic	-	[118]
Fmoc-F_5_-Phe-DAP	NaCl + HC	Water	-	20	33.7	10,776	2273	1	1	-	10–20	Thixotropic Twisted fibers and tapes	Drug delivery	[119]
Fmoc-Tyr	SE	Water/DMSO (98:2 *v*/*v*%)	-	-	4.9	506	59	-	-	-	13	-	-	[95]
	GdL	Water	5.2	<0.1	21.7	10^4^–10^5^	~10^4^	1	1	80	-	Thermoreversible	-	[115]
	HC	PB	7.4	0.47	20	~3000	~800	1	1–10	-	20	Flexible entangled fibers	Antimicrobial activity	[120]
Fmoc-Tyr(PO_4_)	HC	Water	2.5	-	40	~1000	~100	-	-	-	20–25	Bundles (50–100 nm)	-	[121]
	Enzyme	Water	6	-	40	~5000	~2000	-	-	-	20–25	Thermoreversible	-	[121]
Fmoc-Tyr(3NO_2_)	HC	PB	5	2	15.6	~7000	~800	-	-	-	-	No recovery	Antimicrobial activity	[122]
	HC	PB	7	5.6	15.6	~1000	~500	-	-	-	-	Thixotropic	Antimicrobial activity	[122]
	HC	PB	8	11.2	15.6	~1000	~300	-	-	-	-	Thixotropic	Antimicrobial activity	[122]
Fmoc-Tyr /Fmoc-Tyr(Bzl)	SE	Water/DMSO (98:2 *v*/*v*%)	-	-	-	~900	~300	-	-	-	10–50 & 50–80	-	Photothermia Drug delivery	[123]
Fmoc-Phe/Fmoc-Tyr(Bzl)	SE	Water/DMSO (98:2 *v*/*v*%)	-	-	-	~900	~200	-	-	-	10–100	-	Photothermia Drug delivery	[123]
Fmoc-Trp	GdL	Water	5.2	1.9	19	~10^4^	~10^3^	1	10	75	-	-	-	[115]
	HC	PB	7.4	0.03	-	100	10	0.1	1	-	~20	-	Antibacterial	[120]
Fmoc-Met	GdL	Water	5.2	<0.13	27	~10^3^	~10^2^	1	10	-	-	Syneresis	-	[115]
	HC	PB	7.4	0.12	-	1000	100	0.1	1	-	~20	-	Antibacterial	[120]
Fmoc-Gly	GdL	Water	5.2	26.9	33.6	~10^2^	~10^1^	0.1	100	-	-	-	-	[115]
Fmoc-Ile	GdL	Water	5.2	19.8	28.3	~10^2^	~10^1^	1	100	-	-	-	-	[115]
Fmoc-His	Metal	Tris-HNO_3_	9.1	-	10.6	~2000	~100	0.01	0.1	-	~20	-	Antimicrobial activity	[124]
Fmoc-Pro	Metal	Tris-HNO_3_	9.1	-	11.9	~300	~10	0.01	0.1	-	~20	-	Antimicrobial activity	[124]
Fmoc-Ala	Metal	Tris-HNO_3_	9.1	-	12.8	~1000	~100	0.01	0.1	-	~20	-	Antimicrobial activity	[124]
Fmoc-Leu	Metal	Tris-HNO_3_	9.1	-	11.3	~2000	~400	0.01	0.1	-	~20	-	Antimicrobial activity	[124]
Fmoc-Lys-Bct	US	PB	7.4	5	-	~6000	~100	-	-	60	-	Thixotropic	Antimicrobial activity	[125]
Fmoc-Lys(Fmoc)	SE	Water/DMSO (99:1 *v*/*v*%)	6	5	-	~5000	~300	1	1–10	-	-	Thixotropic	-	[126]
	SE	Water/DMSO (99:1 *v*/*v*%)	7.4	5	-	~500	~20	1	10	-	-	Thixotropic	-	[126]
Fmoc-Dap(Fmoc)	SE	Water/DMSO (97:3 *v*/*v*%)	4.9	5.5	-	100	10	10	10	-	150–250	Thixotropic	Drug delivery	[127]
	SE	Water/DMSO (97:3 *v*/*v*%)	7.4	18.2	-	10	1	1	10	-	250–300	Thixotropic	Drug delivery	[127]
	SE	Water/DMSO (97:3 *v*/*v*%)	9.1	23.7	-	1	1	0.1	10	-	250–600	Thixotropic	Drug delivery	[127]
1-NapAc-Phe	GdL	Water	-	-	7.5	941	82	-	-	-	11	-	-	[117]
1-NapAc-3F-Phe	GdL	Water	-	-	7.5	1548	118	-	-	-	20	-	-	[117]
1-NapAc-F_5_-Phe	GdL	Water	-	-	7.5	2522	336	-	-	-	13	-	-	[117]
2-NapAc-Phe	GdL	Water	5.7	15	30	4849	~100	0.1	0.26	45	-	-	-	[116]
2-Nap-Phe	GdL	Water	5.9	19	27	7820	~300	0.1	0.56	48	-	-	-	[116]
Pyr-Phe	HC	PB	7.4	0.85	118.3	~200	~60	-	-	66.4	30–55	Thixotropic Helical fibers	Drug delivery	[128]
Cin-Phe	GdL	Water	4.6	33.9	33.9	2519	~100	1	0.85	41	-	-	-	[116]
Lauroyl-Phe	HC	Water	-	43.2	-	~2000	~100	0.1	0.1–10	-	-	Flat 2D sheets	-	[129]
Bz(4-NO_2_)-Phe	HC	PBS	6	20	20	2000	200	40	100	~40	-	5	Antimicrobial	[130]
BP-Phe	SE	CH_4_/H_2_O	-	2	5	10^2^–10^3^	10^1^–10^2^	1	10–100	-	-	50	Imprinting	[131]
Myr-L-Phe	HC	PB	7	6.7	-	10^2^	10^2^	-	-	37	56	Thixotropic	Enzyme entrapment	[132]
Myr-D-Phe	HC	PB	7	6.7	-	10^2^	10	-	-	37	58	Thixotropic	Enzyme entrapment	[132]

CGC: critical gelation concentration; T_m_: gel–sol transition temperature; G′: storage modulus; G″: loss modulus; Fmoc: fluorenyl-9-methoxycarbonyl; 1-NapAc: 2-(Naphth-1-yl)acetic acid; 2-NapAc: 2-(Naphth-2-yl)acetic acid; 2-Nap: 2-(Naphth-2-yloxy)acetic acid; Pyr: 4-(1-Pyrenyl)butyric acid; Cin: cinnamic acid; Bz: benzyl; BP: 4,4′-dipyridyl; Bct: biocytine; Myr: myristic acid; Lys: lysine; Pro: proline; Gly: glycine; Tyr: tyrosine; Ile: isoleucine; Leu: leucine; His: histidine; Ala: alanine; Met: methionine; Trp: tryptophan; Phe: phenylalanine; Phe(3F): 3-fluoro-phenylalanine; Phe(4F): 4-fluoro-phenylalanine; Phe(4NO_2_): 4-nitro-phenylalanine; Phe(4CN): 4-cyano-phenylalanine; Phe(4NH_2_): 4-amino-phenylalanine; Phe(4CH_3_): 4-methyl-phenylalanine; Tyr(PO_4_): 4-phospho-L-tyrosine; Tyr(3NO_2_): 3-nitro-L-tyrosine; Tyr(Bzl): 4-benzyl-L-tyrosine; Phe(F5): 2,3,4,5,6-Pentafluoro-phenylalanine; PB: phosphate buffer; PBS: phosphate buffer saline; DAP: diaminopropane; DMSO: dimethyl sulfoxide; SE: solvent exchange; US: ultrasound; HC: heating/cooling; GdL: glucono-δ-lactone.

**Table 4:** List of *N*-capped dipeptide gelators, including self-assembly parameters, mechanical properties, melting temperature (T_m_), fibril cross-section, highlights and application. The critical gelation concentration (CGC) and gelator concentration employed in the rheological assays [Gel] are included. The orders (or range) of the limit strain of the linear viscoelastic regime (LVR), critical strain (γ), storage (G′), and loss (G″) moduli are indicated unless the values are detailed in the respective manuscript.

Gelator	Method	Media	pH	CGC (mM)	[Gel] (mM)	G′ (Pa)	G″ (Pa)	LVR (%)	γ (%)	T_m_ (°C)	Fibril (nm)	Highlights	Application	Ref.
Z-FF	SE	Water/HFIP	-	<11.2	22.4	>100,000	<10,000	-	-	-	-	Thixotropic	-	[182]
	SE	Water/AA	-	-	11.2	<100,000	~1000	-	-	-	-	-	-	[182]
	D	Water	-	3.1	-	2000	~150	<10	<30	-	-	-	-	[129]
	HC	Water	-	-	-	300	~30	<10	>100	-	-	-	-	[129]
CBz-FF	pHE	PBS	7.2	0.7	22.4	500	50	-	-	-	1.7	-	-	[190]
C16-FF	D	Water	-	1.8	-	~300	~70	<1	10	-	20 [d]	Helical fibers	-	[129]
	HC	Water	-	-	-	~300	~150	<1	-	-	-		-	[129]
	D	Water/salt	11.7	-	9.1	2361	334	>20	~80	-	-	-		[61]
C14-FF	D/Ca^2+^	Water/salt	11.7	-	9.6	3400	732	<10	~20	-	-	-	-	[61]
Bz4F-FF	HC	Water	7.89	33.5	44.8	5700	~1000	>100-	-	-	8 [d]	T_m_ dependence	Cell growth	[184]
Az-FF	SE	Water/DMSO	3.4	10.2	10.2	200	~30	-	-	-	-	-	Drug delivery	[185]
AzF4-FF	SE	Water/DMSO	7.5	1.8	8.9	1500	~200	-	-	-	-	-	Drug delivery	[185]
Cin-FF	HC	PBS	7.4	4.5	4.5	226	-	-	-	~45	Ribbon	Helical fibers	Cell growth	[187]
In-FF	pHE GdL	Water	-	8.5	21.2	300,000	~5000	-	-	-	100–400 [t]	-	-	[188]
	pHE GdL	Water	4–5	6.4	12.8	100,000	10000	<1	>70	-	-	Fiber dd	-	[189]
NMeI-FF	pHE GdL	Water	4–5	12.4	12.4	200,000	20000	<1	~9	-	-	Fiber dd	-	[189]
Bim-FF	pHE GdL	Water	4–5	4.1	12.3	30,000	2000	<3	~4	-	-	Fiber dd	-	[189]
B-FF	pHE GdL	Water	4–5	2.1	12.7	50,000	4000	<10	~70	-	-	Fiber dd	-	[189]
5H-GL-FF	pHE GdL	Water	-	2	4	~1000	-	-	-	-	-	-	-	[191]
Pyr-YL	pHE	Water	~7.3	-	10	~190	~45	0.1–1	-	-	40–200	Stable ν 0.1–5.0	-	[176]
ThNap-FF	D/Ca^2+^	Water/salt	11.7	-	10	54,944	8786	<1	>10	-	-	-	-	[61]
	SE	Water:DMSO (80:20 *v*/*v*)	~4.3	-	4	~10,000	~1000	>10	-	-	-	Annealing	Molding	[13]
a-FF	pHE	Water	5	20.5	40.9	8090	-	-	1	-	16 [w]	-	-	[192]
g-FF	pHE	Water	5	19.9	39.7	12,613	-	-	0.8	-	15 [w]	-	-	[192]
t-FF	pHE	Water	5	20.9	41.8	6345	-	-	1.2	-	9 [w]	-	-	[192]
c-FF	pHE	Water	5	21.6	43.2	26	-	-	0.6	-	10 [w]	-	-	[192]
Cou-FF	pHE GdL	Water	-	-	9.7	82,000	10,000	-	1	-	42 [d]	-	-	[193]
Fc-FF	D	Water/MeOH (90:10 *v*/*v*)	-	-	5.7	~1000	~40	-	-	-	40–90 [d]	-	-Redox	[198]
BPmoc-FF	D	MES buffer	-	1.0	-	-	-	-	-	43	10–30 [d]	Bundled tape-like	Stim. Resp	[195]
NPmoc-FF	D	MES buffer	-	>0.35	-	-	-	-	-	-	-	-	Stim. Resp	[195]
Bhcmoc-FF	D	MES buffer	-	>0.40	-	-	-	-	-	-	-	-	Stim. Resp	[195]
Nvoc-FF	SE	Water	3.8	-	9	40,000	-	<10	-	-	-	-	Stim. Resp	[45]
Nap-GG	D	Water	~2	3.2	15.8	~500	~40	-	-	46	30 [w]	-	-	[196]
Nap-Ga	D	Water	~2	2.1	15.1	~5000	~450	-	-	51	30, 60 [p]	Left helical	-	[196]
Nap-GA	D	Water	~2	2.1	15.1	~5000	~450	-	-	52	30, 60 [p]	Right helical	-	[196]
Nap-GS	D	Water	~2	2.3	14.4	~5000	~450	-	-	50	50 [w]	-	-	[196]
NapBr-VF	pHE GdL SE	Water Water/DMSO (95/5)	10–12	-	9.5 9.5	27,250 13,710	2610 1870	-	-	-	-	-	-	[197]
NaAc-VF	pHE GdL SE	Water Water/DMSO (95/5)	10–12	-	11.5 11.5	3610 455	495 510	-	-	-	-	-	-	[197]
Fen-VF	pHE GdL SE	Water Water/DMSO (95/5)	10–12	-	10 10	24,250 2460	2530 270	-	-	-	-	-	-	[197]
Pyr-VV	pHE GdL SE	Water Water/DMSO (95/5)	10–12	-	10.1 10.1	25,010 14,250	4760 2640	-	-	-	-	-	-	[197]

CGC: critical gelation concentration; T_m_: gel–sol transition temperature; G′: storage modulus; G″: loss modulus. Amino acids are reported as one letter code. mM = millimol/L; ν = frequency; pHE = pH-exchange (“pH-switch); D = dissolution; [t] = thickness; [d] = diameter; [w] = width; [p] = pitch; Enz = enzymatic deprotection; PB: phosphate buffer; DMSO: dimethyl sulfoxide; SE: solvent exchange; US: ultrasound; HC: heating/cooling; GdL: glucono-δ-lactone. AA: acetic acid; Fiber dd = relationship between fiber dimension and concentrations.
